# Wide‐Bandwidth Nanocomposite‐Sensor Integrated Smart Mask for Tracking Multiphase Respiratory Activities

**DOI:** 10.1002/advs.202203565

**Published:** 2022-08-23

**Authors:** Jiao Suo, Yifan Liu, Cong Wu, Meng Chen, Qingyun Huang, Yiming Liu, Kuanming Yao, Yangbin Chen, Qiqi Pan, Xiaoyu Chang, Alice Yeuk Lan Leung, Ho‐yin Chan, Guanglie Zhang, Zhengbao Yang, Walid Daoud, Xinyue Li, Vellaisamy A. L. Roy, Jiangang Shen, Xinge Yu, Jianping Wang, Wen Jung Li

**Affiliations:** ^1^ Dept. of Mechanical Engineering City University of Hong Kong Hong Kong China; ^2^ Dept. of Biomedical Engineering City University of Hong Kong Hong Kong China; ^3^ Dept. of Computer Science City University of Hong Kong Hong Kong China; ^4^ School of Chinese Medicine The University of Hong Kong Hong Kong China; ^5^ School of Data Science City University of Hong Kong Hong Kong China; ^6^ James Watt School of Engineering University of Glasgow Scotland UK; ^7^ Hong Kong Centre for Cerebro‐cardiovascular Health Engineering (COCHE) Hong Kong China

**Keywords:** Covid‐19, high‐frequency pressure sensors, respiratory sounds recognition, smart masks, sponge structure sensors

## Abstract

Wearing masks has been a recommended protective measure due to the risks of coronavirus disease 2019 (COVID‐19) even in its coming endemic phase. Therefore, deploying a “smart mask” to monitor human physiological signals is highly beneficial for personal and public health. This work presents a smart mask integrating an ultrathin nanocomposite sponge structure‐based soundwave sensor (≈400 µm), which allows the high sensitivity in a wide‐bandwidth dynamic pressure range, i.e., capable of detecting various respiratory sounds of breathing, speaking, and coughing. Thirty‐one subjects test the smart mask in recording their respiratory activities. Machine/deep learning methods, i.e., support vector machine and convolutional neural networks, are used to recognize these activities, which show average macro‐recalls of ≈95% in both individual and generalized models. With rich high‐frequency (≈4000 Hz) information recorded, the two‐/tri‐phase coughs can be mapped while speaking words can be identified, demonstrating that the smart mask can be applicable as a daily wearable Internet of Things (IoT) device for respiratory disease identification, voice interaction tool, etc. in the future. This work bridges the technological gap between ultra‐lightweight but high‐frequency response sensor material fabrication, signal transduction and processing, and machining/deep learning to demonstrate a wearable device for potential applications in continual health monitoring in daily life.

## Graphical abstract

A “smart mask” is developed by using an ultrathin nanocomposite‐based soundwave sensor with high sensitivity and wide‐bandwidth dynamic range, i.e., integrated with machine/deep learning algorithms, the smart mask can identify breath, two‐/tri‐phase coughs, and speaking words directly. The smart mask could be a new wearable Internet of Things device for personal health monitoring and respiratory disease identification tool in the future.

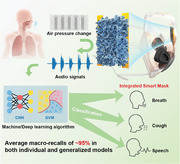

## 1 Introduction

Since the emergence of the coronavirus disease 2019 (COVID‐19),^[^ ^1^ ^]^ it has been generally accepted that universal masking is a necessary measure against the worldwide spread of COVID‐19 because wearing masks can effectively prevent the transmission of coronavirus and influenza viruses from infected individuals.^[^ ^2^, ^3^, ^4^, ^5^ ^]^ Many countries established laws requiring the use of masks,^[^ ^6^, ^7^ ^]^ and wearing masks has become a daily necessity, including as a part of people's social lives. Across the globe, there was a sentiment in early 2022 that the COVID‐19 virus could soon become endemic, similar to common cold flu viruses. However, as warned by A. Katzourakis of Oxford University recently,^[^ ^8^ ^]^ we must set aside optimism and be more realistic about the likely levels of death, disability, and illness that will be caused by a “COVID‐19” endemic phase. It is important to remember that endemicity does not correspond to harmlessness. Malaria, for example, was widely recognized in ancient Greece by the 4th century BC, but it is still considered an endemic disease in 87 countries worldwide, as reported by the Centers for Disease and Prevention (CDC) in 2021.^[^ ^9^ ^]^ In fact, nearly half of the world's population lives in areas at risk of malaria transmission, and malaria caused an estimated 241 million clinical cases and 627 000 deaths in 2020. Moreover, the world must also consider that circulating virus could give rise to new variants, such as the new BA.2 variant (a subvariant of Omicron), which continues to spread across the world.^[^ ^10^ ^]^ Hence, globally, we must use available and proven weapons to continue to fight the COVID‐19 viruses, e.g., wearing masks. In addition, wearing face masks is also an effective way to prevent the spread of other respiratory viruses, such as seasonal human coronaviruses, influenza viruses, and rhinoviruses.^[^ ^2^ ^]^

Therefore, deploying a face mask to monitor human physiological signals has been highly beneficial for personal and public health.^[^ ^11^, ^12^, ^13^, ^14^, ^15^, ^16^, ^17^, ^18^, ^19^, ^20^, ^21^, ^22^, ^23^, ^24^, ^25^ ^]^ Different kinds of smart masks have been proposed to detect body signals, including respiratory rate/heart rate,^[^ ^11^, ^12^, ^13^, ^14^, ^15^, ^16^, ^17^, ^18^ ^]^ skin temperature,^[^ ^12^, ^16^, ^19^, ^20^, ^21^, ^22^, ^23^ ^]^ cough count (based on temperature and pressure change),^[^ ^16^, ^18^ ^]^ blood oxygen^[^ ^12^ ^]^ and airborne pathogens.^[^ ^24^, ^25^ ^]^ Among them, respiratory activities, coughing in particular, are key symptoms of respiratory illness and are usually of great importance for diagnosing diseases, such as pertussis and asthma.^[^ ^26^, ^27^, ^28^ ^]^ Additionally, the latest research also further indicates that the impact of COVID‐19 on the respiratory system may lead to notable changes in the voice of infected people, which can be determined by speaking, breathing, and coughing.^[^ ^29^, ^30^, ^31^ ^]^ Currently, many researchers are collecting respiratory sound audio samples from the public to build AI models and improve machine learning algorithms through smartphone apps and web‐based platforms. However, from a hygiene perspective, coughing or speaking on open surfaces is not desirable, as it may result in further transmission of respiratory diseases. Therefore, it is highly desirable to develop smart masks to monitor respiratory activities, such as breathing, coughing, and speaking.

As one of the most important respiratory activities, breath‐related signals (e.g., heart rate and breath rate) are usually detected by off‐the‐shelf photoplethysmography (PPG) sensors^[^ ^12^, ^13^ ^]^ and thermistors^[^ ^16^ ^]^ integrated with masks. In addition to commercial sensors, advanced nanogenerators and flexible sensors based on new nanomaterials have also been used recently. It is known that breath is the process of air flowing in and out of the lungs, namely, exhalation and inhalation.^[^ ^32^ ^]^ Therefore, it can also be detected as air‐flow‐driven pressure. For example, nanogenerators based on nanofibrous^[^ ^11^ ^]^ and nanostructured polytetrafluoroethylene (n‐PTFE) thin films^[^ ^14^ ^]^ have been used to successfully detect breath activities. An ultrathin pressure sensor with piezoelectric‐like properties was also integrated into a face mask to detect human breath activities.^[^ ^18^ ^]^ For coughing, researchers used integrated sensors to detect the temperature change^[^ ^16^ ^]^ and air flow^[^ ^18^ ^]^ during coughing. These coughing detection sensors focused only on cough counting, and the obtained information would be limited since coughing is a process that consists of several stages. Coughing initiates a series of respiratory activities that cause a sudden explosion of air along with the coughing sound, which usually consists of three phases.^[^ ^33^, ^34^, ^35^, ^36^ ^]^ The first stage is an explosion of the air with a glottal opening producing some noise‐like waveform, and then at the second steady stage when the airflow is decreased, it causes the sound amplitude to also decrease. The third stage is the voiced stage, which is the interruption of the air flow due to the closure of the glottal and periodic vibration of part of the glottis, and it is not always present.^[^ ^36^ ^]^ Therefore, sensors used for cough detection should be sensitive to not only subtle air pressure but also to high‐frequency vibrations.

Another function of the smart mask that draws attention is speech detection. Speech is produced by vocal fold vibration‐induced air vibration when vocal folds come close as the air passes through during the exhalation of air from the lung.^[^ ^37^ ^]^ Recent investigations have demonstrated that both standard surgical masks and N95/KN95 respirators influence the acoustic characteristics of voice,^[^ ^38^, ^39^, ^40^, ^41^ ^]^ attenuating the mean spectral level by 2.0–5.2 dB in the high‐frequency region from 1 to 8 kHz while not considerably affecting the low‐frequency range from 0 to 1 kHz.^[^ ^38^ ^]^ Masks have a greater impact on human speech recognition against a higher level of background noise.^[^ ^42^ ^]^ The high‐frequency components of the human voice provide perceptual information for individual speaker gender^[^ ^43^, ^44^ ^]^ and contribute to speech intelligibility;^[^ ^45^ ^]^ thus, wearing masks causes the dampening of high‐frequency spectral energy, which leads to a decline in clarity of speech, and may consequently affect communication efficiency while interfacing with voice‐recognition devices. For example, mobile phone software that requires voice recognition may not work properly in noisy environments when a person is wearing a mask, and the health monitoring related to the human voice will also be affected. Therefore, integrating a “voice recorder” inside of the mask would be beneficial for speech recognition and understanding when wearing a face mask. Moreover, human speech could also provide health information and potentially be used for disease diagnosis.^[^ ^46^, ^47^, ^48^ ^]^

**Figure 1:**
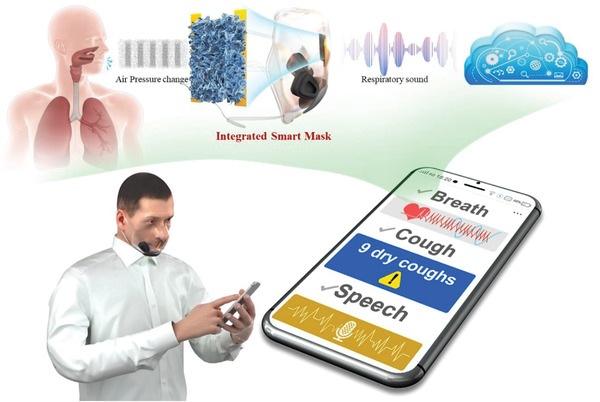
The application of smart masks for monitoring human daily respiratory activities.

Based on the above facts, this work presents the development of a smart mask integrating an ultrathin flexible sponge structure‐based soundwave sensor made of carbon nanotube/polydimethylsiloxane (CNT/PDMS) nanocomposites. A unique double sugar cubes imprint process was used to realize the ultrathin sponge sensing elements that enable high sensitivity in both static and dynamic pressure measurement ranges for tracking, classifying, and recognizing different respiratory activities, including breathing, speaking, and coughing. The 400 µm thick sponge sensing elements show a static pressure sensitivity of approximately 0.79 kPa^–1^ and respond to high‐frequency dynamic pressure generated by the human voice, i.e., sound harmonic energy up to 4000 Hz. Air pressure caused by air movements consisting of air directional flow and air vibration could also be detected, and their frequency composition features were investigated. Various characteristics of the three different respiratory activities were successfully captured. Thirty‐one human subjects were recruited to collect respiratory activities while wearing the smart mask. These data were further processed and classified by support vector machine (SVM) and convolutional neural networks (CNNs). For individual subjects, all 31 human subjects had macro recalls above 90% (with a maximum as high as 100%), and the average reached 95.23% for these three different types of respiratory sounds. The macro‐recall reached approximately 95.88% for the three respiratory sounds among all 31 subjects. Several applications could be developed using this smart mask. For example, as shown in **Figure** **1**, important health‐related signals, respiratory rate (and further heart rate, which is approximately four times the respiratory rate during rest) and cough could be used for the screening and diagnosis of cough‐related diseases.

## 2 Results

### 2.1 Ultrathin, Porous Sponge Structure‐Based Sensor

**Figure 2:**
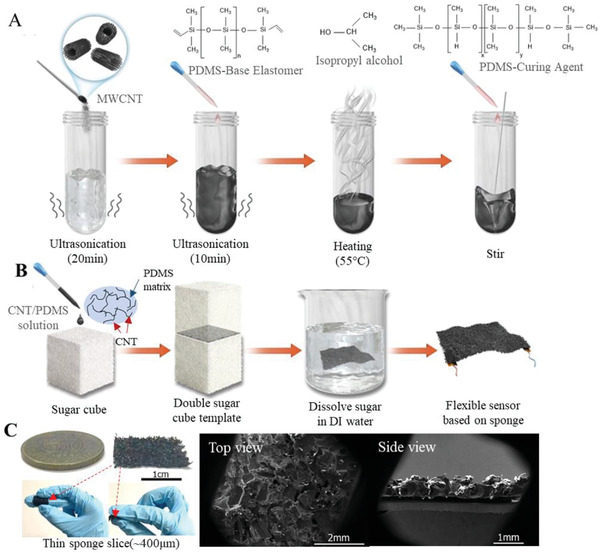
Schematic showing the flexible sponge‐based sensor fabrication. a) The preparation process of carbon nanotube/polydimethylsiloxane (CNT/PDMS) nanocomposites by mixing multiwall CNTs (MWCNTs) with PDMS. b) The fabrication process of a CNT/PDMS sponge using a double‐sugar cube template and a sponge‐based sensor with two soft copper tape electrodes attached to the sponge. c) Optical and scanning electron microscopy (SEM) images of the sponge to show its thin thickness and porous structure.

**Figure** **2**a illustrates the preparation process of the nanocomposites of CNT/PDMS by mixing PDMS with multiwall CNTs (MWCNTs) while isopropyl alcohol (IPA) was applied as the solvent. This mixture solution with CNTs embedded in PDMS matrix could be processed into different structures with templates and curing processes. Solutions with different CNT concentrations (2–5 wt%) were prepared. Figure 2b presents the fabrication of the thin film sponge structure with the CNT/PDMS materials using a novel modified imprint technique with a double sugar cube template. The porous sponge had a minimum thickness of approximately 400 µm (area size of 19.6 × 18.4 mm, the same as the sugar template) with high flexibility (Figure 2c). It has a varied pore size in the range of approximately 150–550 µm. Two pieces of soft copper tape serving as the electrodes were bonded with the thin sponge structure with silver paste to ensure that this sensor could be connected to a circuit to study its electrical properties and sensing performance.

### 2.2 Electrical Properties and the Static Pressure Sensing Performance of the Sensor

**Figure 3:**
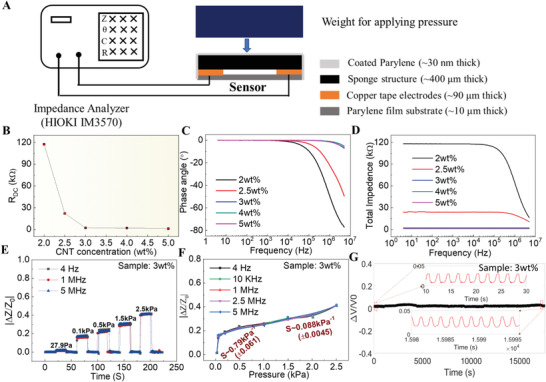
Electrical properties and static pressure sensing performance of the sponge‐based sensor. a) Schematic illustration of the impedance measurement setup. b) Direct current resistance (*R*_DC_) varied with different carbon nanotube (CNT) concentrations of 2, 2.5, 3, 4, and 5 wt%. c,d) Frequency‐dependent (from 4 Hz to 5 MHz) phase angle (c) and total impedance (d) of the CNT/polydimethylsiloxane (PDMS) sponge‐based sensor. Impedance response (e) and the corresponding sensitivity (f) of the sensor with 3 wt% CNT to different pressures under different measurement frequencies (sensitivity results are shown as the mean ± standard deviation). g) Stability performance of the sensor with 3 wt% CNT over ≈8000 cycles (at ≈0.5 Hz).

This kind of synthetic nanocomposite obtained by mixing polymer and nanoconductive materials usually has complex electrical properties depending on the ratio of the conductive materials and cannot be treated simply as a capacitor or a resistor.^[^ ^49^, ^50^, ^51^, ^52^ ^]^ Therefore, it was necessary to study the electrical properties of the prepared CNT/PDMS sponge (≈400 µm) sensors with different CNT concentrations in detail to lay the foundation for their application. **Figure** **3**a shows the experimental setup and Figure 3b–d shows the electrical properties of the CNT/PDMS nanocomposite sponge‐based sensors with different CNT contents from 2 to 5 wt%, which were measured without an applied external pressure. The direct current resistance (*R*_DC_) values of the sensors with different CNT contents are shown in Figure 3b. A higher CNT concentration is associated with a lower resistance of the CNT/PDMS sponge, while the *R*_DC_ exhibited a slight change at CNT concentrations of 3 wt% or higher. Regarding the impedance properties, the changes in the phase angle (*θ*) and the total impedance (*Z*) of the CNT/PDMS sponge‐based sensors at varying frequencies from 4 Hz–5 MHz are shown in Figure 3c,d. At relatively low frequencies, resistive behavior was dominant. The total impedance did not change with frequency, and the phase angle remained near zero. As the frequency increased, both the total impedance and the phase angle changed. At high frequencies, the phase angle was no longer zero, indicating that the sponge had both the resistance component (i.e., the real component of Z) and the reactance component (i.e., the imaginary component of Z). Accordingly, the test frequency range could be delineated into resistive and capacitive regions, which increased and decreased, respectively, with increasing CNT concentration. In addition, the sponges with CNT concentrations of 3, 4, and 5 wt% exhibited similar impedance curves (frequency versus *θ* and *Z*), and they behaved more like a resistor. The formation of conductive networks of CNT in polymer usually follows the percolation theory,^[^ ^53^ ^]^ and the effects of CNT concentration are discussed in Supporting Information. There exists a percolation threshold where the conductivity of the composite would increase significantly, and the value can be considered as the 3 wt% based on the experimental results. The estimated critical exponent value (0.89) of the material developed in this work lies in a reasonable range.

According to the electrical property investigation above, the CNT/PDMS sponge‐based sensor is not composed of solely resistive or capacitive materials. The sensors with 2 and 3 wt% CNT contents have two typical electrical properties. Therefore, the impedance response of the sponge‐based sensor with 2 and 3 wt% contents to pressure was measured. In this study, different static pressures were applied using different weights, and the impedance measurement setup is shown in Figure 3a. Impedance responses to different mechanical pressures were measured at different measurement frequencies to investigate their effects on sensor sensitivity. The total impedance (*Z*) contained a real component (resistance, *Z*_re_ = *Z* × cos*θ*) and an imaginary component (reactance, *Z*_im_ = *Z* × sin*θ*). The response and sensitivity related to these three parameters were measured and calculated, as shown in Figure S1 and Table S1 (Supporting Information). For both sensors, the response curves can be delineated into two linear segments in the pressure ranges of 27.9 Pa–0.2 kPa and 0.2–2.5 kPa with all measurement frequencies. The overall performance of the sensor with 3 wt% CNT concentration is better than that of 2 wt% CNT because it has higher sensitivity in both low‐ and high‐pressure range under all measurement frequencies. The total impedance response of the sensor with 3 wt% CNT to different static pressures is shown in Figure 3e,f. The average sensitivity (i.e., mean value under all measurement frequencies) was determined to be 0.79 (±0.061) kPa^–1^ and 0.088 (±0.0045) kPa^–1^ in the static pressure ranges of 27.9 Pa–0.2 kPa and 0.2–2.5 kPa, respectively. The obtained sensitivity value of the sensor with 3 wt% CNT has little dependance on measurement frequency. Therefore, the nanocomposite sensor which behaved more like a resistor (with a CNT content of 3 wt% or above) was selected for further applications. The sensitivity should be approximately 0.9 kPa^–1^ (the value of *Z*_re_ sensitivity at 4 Hz, as shown in Table S1, Supporting Information) when used in a direct current (DC) circuit. Therefore, the sensor could be applied with a relatively simple measurement circuit (e.g., voltage‐divider circuit) and easily integrated with a microcontroller unit (MCU) board, which usually provides a direct power supply. The stability of the sponge‐based sensor 3 wt% CNT was also tested. As shown in Figure 3g, the stability performance of the sensor was studied with a Mark‐10 Motorized Force Test Stands by applying ≈8000 times (at ≈0.5 Hz) of cycling load on the sensor. The theoretically predicted piezoresistive response of the conductive nanomaterials filled polymer is discussed in Supporting Information. In addition to the CNT concentration, other parameters (e.g., Young's modulus, porosity) would also influence the piezoresistive sensor sensitivity (Figure S2, Supporting Information). The sensor performance could be further improved based on the theoretical analysis.

### 2.3 Acoustic Vibration Frequency Response of the Device

**Figure 4:**
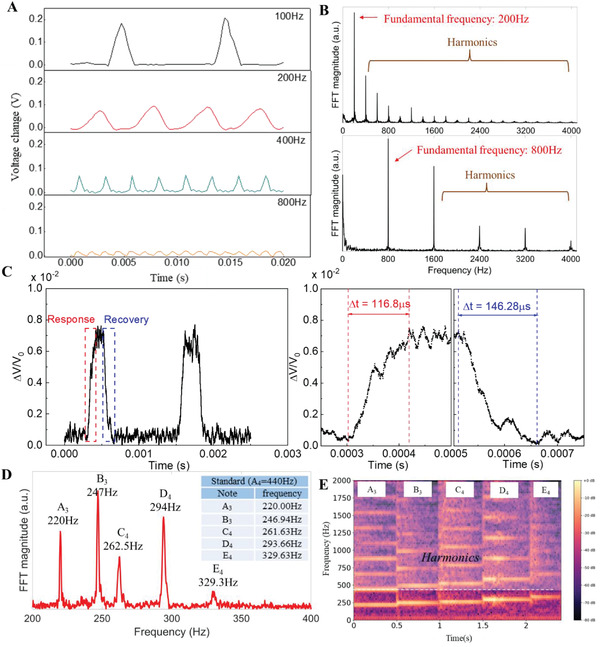
Vibration test of the fabricated flexible sponge‐based sensor using a vibration speaker. a) Signal change at different vibration frequencies of 100, 200, 400, and 800 Hz. b) The corresponding fast Fourier transform (FFT) analysis. (a.u., arbitrary units.) c) Time response (left), and the enlarged view (right) showing the response and recovery time of the sensor. d) FFT analysis when the vibration speaker vibrated at piano sound scales of A_3_, B_3_, C_4_, D_4_, and E_4_ with each sound scale lasting for 0.5 s. e) The corresponding spectrum graph.

The performance of the sponge‐based sensor to high‐frequency vibration was also studied. With the above investigations, the sensor response was measured based on the piezoresistive effect, and a simple voltage divided circuit was applied. The vibration input of 100–800 Hz was provided by a vibration speaker, while the vibration acceleration was measured by a laser Doppler vibrometer (LDV) (the detailed testing setup is shown in Figure S3, Supporting Information). **Figure** **4**a presents the output voltage change when the speaker vibrates at different frequencies, and it shows that the flexible sensor has a good response at high‐frequency vibrations. Since, the fundamental frequency of human speech lies between 80 and 255 Hz for typical adults,^[^ ^54^, ^55^ ^]^ the above results demonstrated that the sponge‐based sensor has a fast enough response to detect human speech generated acoustic signals. The fast Fourier transform (FFT) analysis of the sensor output signal shows that the flexible sensor can detect the fundamental vibration frequency correctly, and harmonics are generated (Figure 4b). The sensor generated narrow and intense spectral peaks with the full width at half maximum (FWHM) of less than 3 Hz at 200 and 800 Hz (as shown Figure S4, Supporting Information), which indicates the sensor can clearly resolve multiple high frequency vibrations. The time response of the sensor was also investigated based on a high‐frequency vibration test, and the result showed a response and recovery time of 116.8 microseconds (µs) and 146.28 µs, respectively (Figure 4c). To further study the frequency accuracy and resolution of the sensor detecting vibrations, Figure 4d shows the frequency components (FFT analysis) when the vibration speaker played with a series of piano scales, i.e., A_3_, B_3_, C_4_, D_4_, and E_3_. Compared with the standard values, the flexible sensor showed good accuracy and resolution when detecting these high‐frequency vibrations. The sensor's output signal was then converted to an audio file using a self‐developed Python program and spectrum analysis was performed based on the audio signals, as shown in Figure 4e. The fundamental frequencies of these notes were clearly detected, as well as the generated harmonics.

### 2.4 Detection of Air Directional Flow and Air Vibration with the Sensor

**Figure 5:**
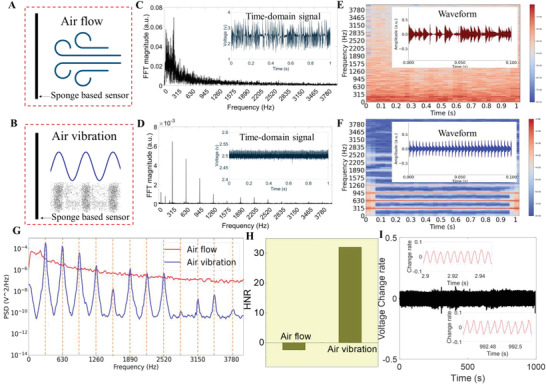
Detection of air directional flow and air vibration with the sponge‐based sensor. a,b) Illustration of the setup of the sponge‐based sensor detecting the (a) air directional flow and (b) air vibration signals. c,d) Fast Fourier transform (FFT) analysis of the output electric signal caused by the c) air directional flow and d) air vibration of 315 Hz. e,f) Spectrograms and waveforms of the e) air directional flow and f) air vibration audios. g) Power spectral density (PSD) plot of the air directional flow and air vibration. h) Harmonic‐noise‐ratio (HNR) value comparison of air directional flow and air vibration. i) Stability characterization of the sensor detecting an air vibration signal of 200 Hz at 76 dB for 1000 s.

The above investigations and results show that the piezoresistive sponge‐based sensor has good sensitivity to both the static applied pressure and the high‐frequency vibration under solid contact. Then, this sensitive flexible sensor was tested to detect the air pressure. Two kinds of air movements were studied. One was the air directional flow, where the air molecules flowed in one direction without moving back and forth. The other was the air vibration, where the air molecules vibrated back and forth at certain frequencies. **Figure** **5**a,b shows the setup schematic illustrations. The sensor was fixed freestanding, and the output voltage signal was varied with the resistance change of the sensor. Figure 5c,d shows the output electric signal and the corresponding generated FFT plots of the air directional flow and air vibration. The results show that the air directional flow caused an irregular output signal during the record time, and no definite frequency component with most energy was in the low frequency range. In contrast, the air vibration caused a stable and regular output signal, and the FFT analysis suggested that the sensor captured the 315 Hz sound signal correctly. The frequency components at multiples of 315 Hz (e.g., 630, 945, 1260 Hz, etc.) were the generated harmonics since it was not a perfect sine wave (the 50 Hz signal and its harmonics were the noise caused by the power supply). The output electric signals were then converted to audio signals since sound actually was the air vibration. More information can be obtained using audio formant. Figure 5e,f presents the audio waveforms and spectrograms of air directional flow and air vibration. The amplitude of air directional flow audio was noise‐like, while the air vibration produced the periodical audio amplitude. The audio spectrogram was the short‐time Fourier transform of the input audio and represents the signal strength or “loudness” of a signal over time at various frequencies present in the waveform, which could also show that the energy levels vary over time. The spectrogram showed that the directional airflow mainly caused low‐frequency energy and varied with time. However, the energy of the air vibration caused signal focused on the 315 Hz and its harmonic frequencies (i.e., 630, 945, 1260 Hz…). In addition, the energy at these frequencies remained stable over time, which was an important feature that differentiated it from the air directional flow signal. Another parameter, power spectral density (PSD), which represented the spectral energy distribution per unit time, showed that the energy of air directional flow decreases steadily with the increasing frequency, while the air vibration presented several peaks (315 × *n* Hz, *n* = 1,2,3…) over frequency (Figure 5g). The harmonic‐to‐noise ratio (HNR) measured the ratio between periodic and nonperiodic components of the audio, as shown in Figure 5h. Air directional flow had a negative HNR value since it had almost no periodic component, while air vibration had a positive HNR value, as it was the periodic signal. In summary, the air directional flow and the air vibration caused signals were different in the aspects of both the time domine and the frequency domine. The stability of the developed device due to high‐frequency air vibration source was studied by detecting an audio signal of 200 Hz and 76 dB for 1000 s as shown in Figure 5i, i.e., the sensor was induced to vibrate for 200 000 times in total. The limitation for sound detection was also investigated and the result is shown in Figure S5 (Supporting Information). The result indicates that the device can detect 200 Hz sound of about 58 dB, which is close to the human normal conversation sound level (i.e., about 60 dB).^[^ ^56^ ^]^ In addition, the device can also detect air vibrations of at least 800 Hz, as shown in Figure S6 (Supporting Information).

### 2.5 Smart Mask Based on the Flexible Sponge‐Based Sensor

**Figure 6:**
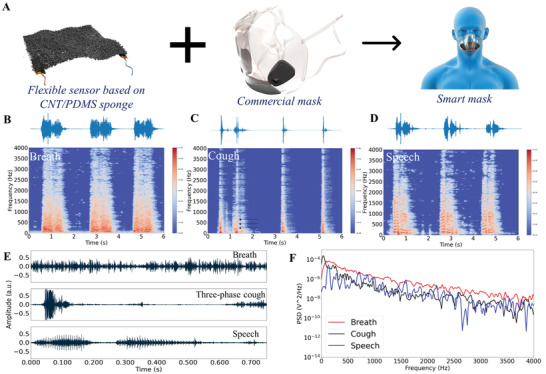
Smart mask to detect the human respiratory activities of breath, cough, and speech. a) Illustration of the integrated smart mask. b–d) Representative waveforms and spectrograms of the audio signals of b) breath, c) cough, and d) speech. e) Individual detailed waveform of the three kinds of respiratory activities. f) Power spectral density (PSD) plot of the three activities of breath, cough, and speech.

The flexible thin sponge‐based sensor has been demonstrated to have a high sensitivity regardless of static pressure and dynamic pressure. It could also detect air movements, including air directional flow and air vibration. Therefore, the idea of integrating this sponge‐based sensor into a commercial mask to measure human daily respiratory activities was proposed. As shown in **Figure** **6**a, the sensor was fixed in front of the inside of the mask and kept freestanding to detect human breath, cough, and speech. Based on the production mechanism of breath,^[^ ^32^ ^]^ cough,^[^ ^36^ ^]^ and speech,^[^ ^37^ ^]^ the breath mainly involves the air directional flow process, while cough involves both air directional flow and air vibration process, and speech mainly involves an air vibration process along with some air directional flow. Therefore, these three respiratory activities should be detected by our sponge‐based sensor integrated smart mask (see Movie S1, Supporting Information).

Figure 6b–d shows the waveforms and spectrograms of breath, cough, and speech signals detected by the developed integrated smart mask. Figure 6e presents the single clean signals of breath, three‐phase cough, and speech (robot). The breath signal is similar to the air directional flow signal, which has an irregular amplitude and mostly low‐frequency energy considering a single clean breath signal. For a continuous measurement signal (Figure 6b), breath counting over time (breath rate) can also be obtained. The recorded cough signals consist of two‐ and three‐phase cough, where the third phase of the three‐phase cough has vibration features (short‐time stability of frequency), as shown in Figure 6c. Similarly, coughs can be counted over time considering the continuous measurement. The speech signal shows obvious periodical vibration features with typical short‐time stability and harmonics. Figure 6f provides the PSD curves of the three respiratory activities (i.e., breath, cough, and speech). The breath's PSD decreased steadily with the increasing frequency, while for the speech signal, the value varied greatly and showed substantial peaks when decreasing with frequency. Cough performed between the breath and speech. The signal had high energy at a very low frequency and then decreased as the frequency increased, with some obvious fluctuations. These results proved that the breath was detected as the air directional flow and speech was mainly detected as the air vibration, while the cough had the features of both air directional flow and air vibration. In addition, the audio of the speech was heard to be the “robot,” which indicates that our sensor could sense subtle air vibrations. To make our developed smart mask portable and applicable, an ESP32 (a commercial MCU board) based wireless module was designed and prepared to sample the output signal and to transmit the data to a computer via the Wi‐Fi protocol. The test results (Figure S7, Supporting Information) showed that similar signals of breath, cough, and speech could be obtained using this self‐developed wireless device compared with the data recorded by the oscilloscope.

### 2.6 Recognition of Three Respiratory Activities with the Integrated Smart Mask

**Figure 7:**
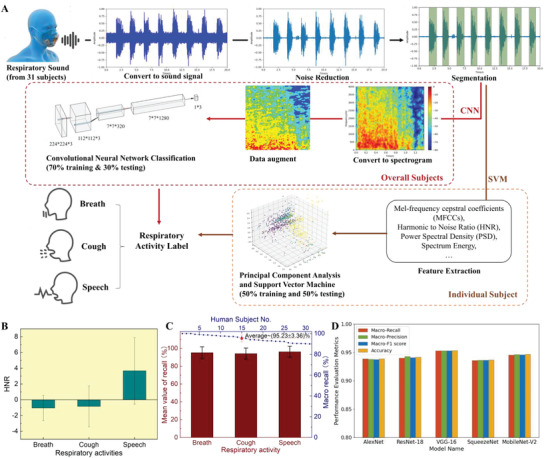
Data processing and classification results. a) Data processing flow of support vector machine (SVM) and convolutional neural networks (CNN) classification. b) The mean harmonic‐to‐noise ratio (HNR) value of breath, cough, and speech for 31 human subjects. The results are shown as the mean HNR ± standard deviation (*n* = 1047). ^∗∗∗^ *p* < 0.001, as determined by one‐way analysis of variance (ANOVA). c) The mean value of the recall from the 31 individual subjects with SVM classification. Error bars represent the differences between datasets of different people. d) Results comparison among five CNN models for the general dataset of all 31 subjects.

To demonstrate our integrated smart mask's capability to monitor people's daily respiratory activities, 31 human subjects were randomly recruited to perform breathing, coughing, and speaking (say “robot”) while wearing the smart mask to collect data. The basic information (e.g., age, gender, and native language) on these human subjects is presented in Table S2 and Figure S8 (Supporting Information). **Figure** **7**a shows the data processing flow, where the recorded change of voltage signals was converted to audio files first and the segmentation work was performed manually based on the visualized waveform and spectrogram. The statistics for the HNR values from the 31 subjects are shown in Figure 7b. The results suggested that speech had the highest and a positive HNR value, meaning that most speech signals obtained were effective since they had vibration features. The mean HNR value of breath was the lowest and negative because the breath signal had almost no periodical vibrations. For cough, the mean HNR value was between the speech and breath signals, which was reasonable because the first two phases of cough induce air flow, while the third phase was the voiced phase, but not all coughs presented this phase. The statistics for the data from 31 human subjects showed consistency with the typical breath, cough, and speech signals in the main features, which suggested that our developed sponge‐based smart mask had good stability and robust performance. To meet different needs, custom models for each subject were built with the SVM algorithm, which performed well even with small data based on individual datasets, while a more general model for all subjects was built with the CNN algorithm, which was more suitable for large data based on the overall dataset. That way, the system could either be used for personal health management or community monitoring. Fifty‐three features (see Table S3, Supporting Information), including HNR and PSD were extracted for SVM classification. Each individual subject had an average of 108 pieces of data in the dataset consisting of breath, cough, and speech signals. Based on the classification results, the macro‐recall (arithmetic mean of the recall value for all the classes) and the recall of each class (breath, cough, and speech) were calculated. Figure 7c shows the mean value of the recall of 31 human subjects, where the mean recall value for each class is above 94% (i.e., 95.22%, 94.18%, and 96.29% for breath, cough, and speech, respectively). In addition, all 31 subjects had a macro‐recall above 90% (the maximum was as high as 100%), while the mean macro‐recall value was approximately 95.23% (±3.36%). Further studies on building a more general model with the CNN method, which was also commonly used to analyze respiratory sounds with the input of spectrograms or Mel‐spectrograms and had a good recognition accuracy for tasks of disease recognition,^[^ ^30^, ^31^ ^]^ were carried out. The processing flow is shown in Figure 7a, and more detail can be found in the Experimental Section. We trained five common CNN models in our dataset, containing three classic CNN models (i.e., AlexNet, ResNet‐18, and VGG‐16) and two lightweight CNN models (i.e., SqueezeNet and MobileNet‐V2). The classification results are shown in Figure 7d and Table S4 (Supporting Information). The highest classification macro‐recall (i.e., 95.88%) was achieved in the VGG‐16 model on the dataset of all 31 human subjects, and MobileNet‐V2 also obtained 94.54% macro‐recall with much fewer model parameters which could be used in mobile phones.

## 3 Discussion

Pressure sensors have long been used for health monitoring^[^ ^57^, ^58^, ^59^ ^]^ and other biological related studies.^[^ ^60^, ^61^ ^]^ More recently, by integrating with low‐power wireless technologies, pressure sensing systems have become more convenient to use while also improved user‐comfort and practicality.^[^ ^62^ ^]^ Monitoring human daily respiratory activities is a promising application for acoustic pressure sensors. Therefore, this research demonstrated a smart mask with integrating a nanomaterial‐based high‐frequency response acoustic pressure sensor to monitor human respiratory activities of breathing, coughing, and speaking. The flexible acoustic pressure sensor was fabricated based on CNT/PDMS ultrathin sponges with excellent sensitivity in both static and dynamic pressure input, so this flexible pressure sensor had a wide application in the monitoring of various human physiological signals, whether at a low frequency or a high frequency. Therefore, the respiratory activities of breath, cough, and speech could all be detected using the same ultralightweight sensor. To be applied inside the mask, the sensor should also be harmless to the human body and work stably in high humidity environments. The sensing material (i.e., CNT/PDMS nanocomposites) proved to be biocompatible, and no free CNTs were observed during use.^[^ ^63^ ^]^ A layer of 30 nm thick parylene‐c, which is also biocompatible^[^ ^64^ ^]^ and resistant to water vapor,^[^ ^65^ ^]^ was coated on the surface of the sensor to make it work well even in a humid environment and further prevented free CNTs from falling off. Figure S9 (Supporting Information) shows the contact angle measurement with the liquid/volume of water/≈5 µL on the sponge‐based sensor. Therefore, this developed flexible sensor can be integrated inside the mask to detect respiratory activities that are accompanied by the safe production of water vapor.

Human respiratory activities mainly involve airflow and air vibration. To monitor respiratory activities at a certain distance from our mouth or nose, the developed sensor was verified for its ability to sense different air movements (i.e., air directional flow and air vibration). For smart mask preparation, the sensor should be kept freestanding between the mask's inner surface and the human face and fixed inside the mask for better detection, which can also help effectively avoid the sound attenuation effect of the mask in the high‐frequency region (i.e., above 1 kHz). Experiments on human subjects wearing this smart mask showed that the smart mask could detect and differentiate three common respiratory activities of breath, cough, and speech. The breath signal was mainly caused by directional air flow, while the cough signal included both directional air flow and air vibration (when the third voiced phase occurred). After converting these signals to audio sound (Movie S2, Supporting Information), the audio corresponding to breath was a noisy sound since only air directional flow was induced without air vibration, which caused a meaningful sound. A coughing sound can sometimes be heard in the third, voiced, phase because it would induce air vibration. It is suggested that not only can cough counting be recorded, but the details of the cough signal can also be obtained and analyzed using this smart mask.

The speech was detected mainly due to the air vibration generated, and thus, the corresponding audio could be clearly recognized. In addition to “robot,” different words (including both English and Chinese) were also recorded and can be recognized (see Movie S3, Supporting Information). The generated waveforms and spectrograms of the different words are shown in Figure S10 (Supporting Information). Although air‐conducted human voices have high‐frequency harmonics of thousands of Hertz, information up to 3400 Hz should be enough for communication since this is the upper limit of the telephone bandwidth used today.^[^ ^66^ ^]^ The spectrograms in Figure S10 (Supporting Information) show that the sensor can detect information of approximately 4 kHz. Therefore, this smart mask has great potential to help improve speech recognition intelligence, which is degraded by the barrier of face masks. However, there are still some air flows that “impact” the sensor, so the speech sounds contain a “popping sound,” similar to when a speaker is too close to a microphone. Applying a blowout hood may help reduce this popping noise and improve the audio quality. Flexible acoustic sensors have been investigated in the past, including capacitive,^[^ ^54^ ^]^ piezoelectric,^[^ ^67^, ^68^ ^]^ triboelectric,^[^ ^69^ ^]^ and piezoresistive sensors.^[^ ^70^, ^71^, ^72^ ^]^ Compared with capacitive, piezoelectric, and triboelectric sensors, piezoresistive acoustic sensors have the advantages of low‐cost, easy fabrication and integration, and single layer structure.^[^ ^72^ ^]^ Capacitive sensors typically have a resonant frequency above audible sound so they exhibit a flat frequency response.^[^ ^54^ ^]^ However, understanding the mechanical resonant characteristics of the designed sensor is very important for other types of sensors due to concerns for relatively low sensitivity for frequency band below the resonant frequency and the drastic degradation of sensor response after the resonant frequency.^[^ ^68^ ^]^ For example, prior work showed that, although some signal distortions were observed close to the device resonant frequency,^[^ ^72^, ^73^ ^]^ the material and mechanical design of acoustic devices could be investigated to use the high sensitivity sensor response around the resonant frequency.^[^ ^68^, ^72^ ^]^ Multiresonant sensors have also been studied to obtain high response sensitivity in a wide detection range.^[^ ^67^, ^72^ ^]^ Piezoresistive acoustic sensors are usually fabricated as crack‐based structures, including microcrack,^[^ ^71^ ^]^ nanocrack,^[^ ^70^ ^]^ and point crack,^[^ ^72^ ^]^ while the three‐dimensional (3D) sponge structure based acoustic sensors (as presented in this work) were rarely studied before. Nevertheless, sponge‐based sensors were applied to detect mechanical vibration signals in the past.^[^ ^74^, ^75^ ^]^ Also, another past work showed that a 3D graphene foam structure exhibited a resonant frequency of below 100 Hz, and the resonant frequency could be tuned with varying in the material, structural parameters, and support methods (i.e., boundary conditions).^[^ ^76^ ^]^ For the CNT/PDMS nanocomposite sponge structure sensor in this work, its resonant frequency is related to multiple factors such as the device shape, material modulus, and porosity, as discussed in the Text S2, Supporting Information (consider it as a simply supported rectangular plate). Experimentally, the sensor was mounted on a rigid bulk substrate and a vibrational frequency sweep from 1 to 400 Hz (increase by 1 Hz per second) with an actuator was performed. As shown in Figure S11 (Supporting Information), the first resonant frequency of the sensor is about 53 Hz. However, since the mounting method of the sensor can also affect its resonant frequency,^[^ ^76^ ^]^ the resonant characteristics of the sensor integrated in the smart mask, where it was kept freestanding, should vary from the value obtained from the above experiment. On the other hand, the indentation modulus (obtained using microhardness test) of the CNT/PDMS nanocomposite increases with increasing CNT concentration (Figure S12, Supporting Information). Therefore, the resonant frequency of the CNT/PDMS sponge sensor can be designed by adjusting the material and the structural parameters. Theoretically, based on the Equation S12 and Figure S12 (Supporting Information), the resonant frequency of the CNT/PDMS sponge structure sensor would increase with increasing CNT concentration, increasing sponge thickness, and decreasing sponge porosity. The result is similar to the previous work on the 3D graphene foam.^[^ ^76^ ^]^ In addition, the parameters discussed above could also affect the pressure sensitivity of the sensor (Equation S5 and Figure S2, Supporting Information). Therefore, although the fabricated sponge structure sensor can already detect speeches currently, the sensor can be further improved (e.g., designed with multiresonant frequency characteristics) to have more superior performance in acoustic sound detection.

The results of the developed smart mask based on rigid masks detecting human breath, cough, and speech suggest that their distinguishable features can be identified so that different respiratory activities can be recognized using our classification algorithm. Since the most used mask currently is the deformable mask based on polypropylene nonwoven fabric, the fabricated CNT/PDMS sponge structure sensor was also tested by integrating it with a commercial deformable face mask (Movie S4, Supporting Information). The results (Figure S13, Supporting Information) show the respiratory sounds of breathing, coughing, and speaking (i.e., “robot”) can be successfully detected even if the mask is flexible. The harmonic features of the speech signal can also be recognized although its response amplitudes (as well as for breath signals) are lower than that for a sensor fixed to a rigid mask. Hence, the sensor's applicability in the more commonly used deformable commercial masks could be assessed in the future by performing long‐term stability tests and also their consistency in collecting respiratory sound data from multiple subjects.

## 4 Conclusion

In conclusion, a flexible acoustic wave sensor was fabricated based on CNT/PDMS composite sponges with a modified imprint technique to make it as thin as 400 µm. The sensor (3wt% CNT content or above) performed well with the piezoresistive mechanism according to the electrical properties and the sensitivity investigation, which showed 0.79 kPa^–1^ and 0.088 kPa^–1^ in the pressure ranges of 27.9 Pa–0.2 kPa and 0.2–2.5 kPa, respectively. The flexible sensor was also tested to detect the dynamic pressure of the fundamental frequency of 100–800 Hz, which showed a great performance in frequency accuracy, harmonics detection (up to 4000 Hz), and resolution for vibration detection. In addition, this flexible sensor showed the ability to sense air movements, including air directional flow and air vibration, which had different characteristics (represented by features used in classification). Moreover, air directional flow caused relatively irregular signals, with energy mainly focused in the low frequency range, while air vibration caused a periodical signal, with energy focused in the vibration frequencies (fundamental frequency and the corresponding harmonics). These results showed that the flexible sensor could be used to detect human respiratory activities by integrating with a commercial polycarbonate mask. It was demonstrated that our developed smart mask could detect and differentiate three common respiratory activities, including breathing, coughing, and speaking. The classification result of the three activities suggested an average macro‐recall of approximately 95.23% (with an individual dataset of 31 human subjects) and 95.88% (with a dataset containing all 31 human subjects), which indicates the proposed entire system can be applied in both personal and public health monitoring and management.

## 5 Experimental Section

### Preparation of the CNT/PDMS Nanocomposites

The preparation process for the CNT/PDMS composite solution involved mixing multiwall CNTs (MWCNTs; XFNANO, China) and PDMS (Sylgard184; Dow, USA) in IPA (Anaqua Chemical Supply, Hong Kong). IPA was used as the solvent because both CNT and PDMS are partially soluble in it.^[^ ^63^, ^77^ ^]^ The MWCNTs had diameters of 10–20 nm and lengths of 10–30 µm (provided by the manufacturer). First, MWCNTs were dispersed in a sufficient quantity of IPA and ultrasonicated for 20 min to obtain a dispersion of CNTs. Then, PDMS‐base elastomer was added to the dispersion and ultrasonicated for 10 min. Subsequently, the mixture was placed on a hotplate (IKA, Germany) maintained at 55 °C to completely evaporate the IPA. Thereafter, PDMS‐curing agent (the weight ratio of PDMS‐ base elastomer and PDMS‐ curing agent is 10:1) was added to the solution and was mechanically mixed. Finally, air bubbles were removed from the mixture through vacuum treatment. In this manner, CNT/PDMS composites with CNT concentrations of 2, 2.5, 3, 4, and 5 wt% were prepared by mixing 0.2/0.25/0.3/0.4/0.5 g of MWCNTs with 10 g PDMS.

### Preparation of the CNT/PDMS Sponge

CNT/PDMS sponges were fabricated using sacrificial sugar cubes (Taikoo; purchased from a supermarket in Hong Kong) measuring 19.6 × 18.4 × 11.7 mm (thickness). The CNT/PDMS composite solution was dropped (with a syringe) onto the surface of a sugar cube and spread smoothly across its surface. Then, a second sugar cube was placed atop the first cube and cured in an oven at 70 °C for approximately 2 h. Finally, the remaining sugar was dissolved in deionized water to obtain a CNT/PDMS sponge sheet. This modified imprinting technique was used to fabricate sponges with a very thin thickness of approximately 400 µm. Sponge samples with different CNT weight concentrations (2–5 wt%) were prepared from the same batch of sugar cubes, and all samples were made the same thickness by controlling the solution amount.

### Fabrication of the CNT/PDMS Sponge‐Based Pressure Sensor

Then, flexible pressure sensors were fabricated based on the thin film sponge. Two sheets of copper tape served as the electrodes and were bonded to electrical wires. The sponge was attached to the electrodes using conductive silver paste, after which the paste was solidified using a hot air gun (Saike, China) at 100 °C for approximately 1 min. A piece of parafilm served as a substrate when needed. The entire sensor could also be packaged by coating a layer of parylene‐c thin film on the surface. The coating process was applied under 17 millitorr, the pyrolysis temperature was 690 °C, the vaporization temperature was 175 °C, and the deposition temperature was room temperature. The resulting thickness was approximately 30 nm when 0.1 g parylene‐c was applied.

### Characterization of the CNT/PDMS Sponge‐Based Sensor

The porous structure of the CNT/PDMS sponge was characterized using scanning electron microscopy (SEM, FEI Quanta 450). Direct current resistance was measured using a digital multimeter (Fluke 15B+, USA), and impedance characteristics were recorded using an impedance analyzer (HIOKI IM 3570, Japan) in the 4 Hz–5 MHz range at 1 V. A series of static pressures from 27.9 Pa to 2.5 kPa were realized by applying different weights. The changes in impedance under different frequencies (4 Hz, 10 kHz, 1 MHz, 2.5 MHz, and 5 MHz) were recorded using the impedance analyzer at a sampling rate of 1 Hz. Sensitivity was calculated using the equation (i.e., the slope of the curve):

(1) $$S=\frac{\Delta Z/Z_{0}}{P-P_{0}}$$

where ΔZ represents the change in impedance (or its real/imaginary part) in response to external pressure *P*, and *Z*_0_ is the initial impedance (or its real/imaginary part) of the pressure sensor. The cycle test was performed using the Motorized Force Test Stand (Mark‐10, Series ESM) with an up‐and‐down speed of ≈20 mm min^−1^ for ≈8000 cycles. For the vibration test, the flexible sensor was attached to a vibration speaker (a commercial product of JBL Plus 3), and the speaker was controlled to vibrate at different frequencies by playing the corresponding audio. Meanwhile, a laser Doppler vibrometer was applied to measure the actual vibration frequency of the speaker. The measurement voltage‐divider circuit contained a DC power source (5 V) and a voltage‐divider resistor, which was used in the cycle test (Figure 3g), and vibration test (Figure 4). The change in the output voltage signal of the sensor was recorded using an oscilloscope. Finally, the sensor was fixed to apply air directional flow and air vibration from a distance. The two pieces of electrodes of the sponge‐based sensor were fixed to two separate pieces of glass with some distance to make the sensing area freestanding. A low‐cost, micro power consumption microphone, MAX4466, was used in this test, and its circuit is shown in Figure S14 (Supporting Information). MAX4466 is a mature product that was integrated with the electret microphone. The developed sponge‐based sensor can be easily integrated into this amplifying module by replacing the electret microphone and worked well. The air directional flow pressure was provided by an air gun blowing air to the sensor with constant velocity, and the air vibration was induced by the speaker playing a 315 Hz sound in front of the sensor. The sound level was measured with a sound level meter integrated on the Apple Watch SE, which was calibrated with a sound level calibrator (ANDTEK, ND9A). The indentation modulus (*E*_IT_) of the materials was measured using the Micro‐hardness Tester (Fischer/Fischerscope HM200 XYp) with a load of 50 mN and holding time of 10 s. The resonant frequency was investigated by applying an actuator (Gelsonlab Hspw‐003) giving a vibration force upright to the sensor with a rigid bulk metal substrate and frequency increasing at 1 Hz per second from 1 Hz to 400 Hz.

### Smart Mask Preparation and Human Respiratory Activity Detection

The developed thin film sponge‐based sensor was then fixed to the inner side of a commercial transparent mask. The commercial polycarbonate mask used in this project had a hard texture (with a Young's modulus of approximately 2.4 GPa for polycarbonate^[^ ^78^ ^]^) and could maintain a fixed shape with three filters located on the two sides of the cheeks and the chin, as shown in Figure 1. The deformable mask tested in Figure S13 (Supporting Information) was a commercial face mask made of polypropylene spun‐bond non‐woven fabric (Manning, Hong Kong). The sensor was fixed to the approximate position of the mouth and nose of the mask. First, the two pieces of sensor electrodes were attached to two pieces of PDMS strip, which had a similar area of the electrodes and was approximately 2 mm thick with double‐sided tape. Then, the sensor was fixed to the inside of the mask by attaching the two pieces of PDMS to the mask with double‐sided tape to make the sponge sensing area flat and freestanding. A wireless data acquisition board was also dedicated based on ESP32, which was a feature rich MCU with integrated Wi‐Fi and Bluetooth connectivity. It collected data with an 8 kHz sampling rate and transmitted data to a computer via Wi‐Fi. This homemade ESP32‐based wireless module had a size of 31 x 22 x 14 mm and a weight of 13 g (with a battery). The power consumption of this device was low, where the current was 56 mA at idle status (i.e., the device is standby and ready for data collection) and 140 mA at working status. Volunteers were asked to wear the mask naturally and perform the respiratory activities of breathing, coughing (voluntary), and speech (i.e., speaking “robot”). The experiments were approved by the Human Subjects Ethics Sub‐Committee of City University of Hong Kong (Reference NO. 2‐2021‐52‐F) and written consent forms were obtained from all the participants. Six pieces of data were obtained for each human activity, while each piece of data lasted for 40 s and consisted of at least five breath/cough/speech signals. The human subjects consisted of males (11) and females (20), and the age range was between 22 and 31 years. Signals were acquired with the circuit based on the MAX4466 audio preamplifier module, and they were sampled by both the oscilloscope and the self‐developed wireless board. The following data analysis process was based on the data acquired from the two methods.

### Data Processing and Classification

Signals of human respiratory activities were converted to audios by a self‐developed program, followed by noise reduction work. A segmentation process was performed manually to select the clean signals for each activity from the noise according to the visualized waveform and spectrogram. Fifty‐three features (shown in Table S3, Supporting Information) were extracted for the SVM^[^ ^79^ ^]^ classification process. Before the final classification, principal component analysis (PCA) was used to reduce the dimensionality and compute the main components of all the features. The SVM algorithm was applied to perform the classification (50% data for training and 50% for testing), and the metric recall was selected to evaluate its performance. Recall predicts the correct proportion of all samples that are actually positive, and the recall for activity *i* is defined as follows:

(2) $${\mathrm{Recall}}_{i}=\frac{N_{\mathrm{TP}}\left(i\right)}{N_{\mathrm{TP}}\left(i\right)+N_{\mathrm{FN}}\left(i\right)}$$

where *N*_TP_(*i*) is the number of the true predict case of activity *i*, and *N*_FN_(*i*) is the false negative error number of activity *i*. A high recall value indicates that most of the behavior samples are correctly classified. Due to the size differences between the three activities, the average recall value (macro‐recall) was adapted as the main metric to represent the model's overall performance, which is expressed as follows:

(3) $$\mathrm{Macro}-\mathrm{recall}=\frac{1}{N}\sum_{i=1}^{N}{\mathrm{Recall}}_{i}$$

where *N* is the total number of activities.

CNNs^[^ ^80^ ^]^ were also applied with the spectrogram picture as the input. The spectrograms were generated after the segmentation process. To obtain the input spectrograms with the same size, the segmented audio signals of different lengths were resampled, and spectrograms of size 256 (pixels) × 256 (pixels) × 3 (RGB channels) were generated. Several data augmentation techniques, including random cropping, normalization, and random rotation, were applied to the spectrograms. Seventy percent of the spectrogram images were input into the CNN models as the training dataset, and the others were input as the testing dataset. Five common CNN models were trained in the dataset, containing three classic CNN models (i.e., AlexNet, ResNet‐18, and VGG‐16) and two lightweight CNN models (i.e., SqueezeNet and MobileNet‐V2). The cross‐entropy loss function and the Adam optimization approach were used. Appropriate learning rates for 200 epochs were used for these models. The evaluation metrics of recall was calculated as Equation (2), and the precision and F1‐score for activity *i* were calculated as Equations (4) and (5), respectively:

(4) $$Precision_{i}=\frac{N_{\mathrm{TP}}\left(i\right)}{N_{\mathrm{TP}}\left(i\right)+N_{\mathrm{FP}}\left(i\right)}$$

(5) $$F1-{\mathrm{score}}_{i}=\frac{2\times {\mathrm{Precision}}_{i}\times {\mathrm{Recall}}_{i}}{{\mathrm{Precision}}_{i}+{\mathrm{Recall}}_{i}}$$

where *N_FP_* (*i*) is the false positive error number of activity *i*. The average precision and F1‐score values were calculated similar as the Equation (3). The metrics presented in Figure 7d are the average values of the activities. And accuracy can evaluate the overall performance which was calculated as Equation (6):

(6) $$\mathrm{Accuracy}\left(\mathrm{total}\right)=\frac{N_{\mathrm{TP}}\left(\mathrm{total}\right)}{N_{\mathrm{total}}}$$

where *N*_total_ is the total number of the testing set. The accuracy is highly affected by the ratio of samples between different behaviors.

### Statistical Analysis

All shown data are representative for the samples. The sample size for the sensor characterization is provided in the subsection entitled “Characterization of the CNT/PDMS Sponge‐Based Sensor”. The sample size for the human test is provided in the subsections “Smart Mask Preparation and Human Respiratory Activity Detection” and “Data Processing and Classification” in the Experimental Section. More detail information was also provided in the Figure captions. The statistical data plotting were performed using OriginLab and Matlab software. Data in Figure 3b,f were presented as mean ± standard deviation. Data in Figure 4a were presented with a moving average to smooth the curve. For the respiratory sound collected from the human subjects, each analog signal was under‐sampled and converted to a waveform audio file (bit rate equals 1411kbps). The data were normalized such that all values lie between 0 to 65 535. Then, the clean respiratory signals (i.e., breath/cough/speech) would be extracted manually from the audio file after noise reduction. The total number of samples was *N* = 3368, so the average sample size for each subject was ≈108. Bar plots in Figure 7b,c are expressed as the mean ± standard deviation. And, a one‐way analysis of variance (ANOVA) is implemented to analyze the difference in the HNR values between three signal groups (Figure 7b). Figure 7d displays the best performance of the five CNN models in classifying three signal groups. In the final classification evaluation based on CNN, some common metrics (i.e., recall, precision, F1 score, and accuracy) were used to analyze the classification models. These statistical analyses of classification were performed using Python 3.8.10, and CNN training and evaluation were completed using an open‐source machine learning framework (PyTorch v1.12.0).^[^ ^81^ ^]^

### Ethics approval statement

Human Subjects Ethics Sub‐Committee of City University of Hong Kong gave ethical approval for the human test in this work (Reference No.: 2‐2021‐52‐F).

## Conflict of Interest

The authors declare no conflict of interest.

## Author contributions

W.J. L. and H.Y.C. supervised and guided the project. J.S., Y.L., and C. W. contributed equally to this work. W.J.L. conceived the initial concept and C.W. carried out the initial background review. W.J.L., H.Y.C., and J.S. conceived the device and experimental design. M.C. and G.Z. developed the circuit and wireless board. Y.L., K.Y., X.C., W.D. helped recruited the human subject volunteers. J.S. performed the experiment and analyzed the data. Y.L. performed the classification work with algorithms, with J.S. providing some help. M.C., Q.H., Q.P., Z.Y., and Y.C. assisted in part of experiments and data analysis. J.S., Y.L., C.W., H.Y.C., and W.J.L. co‐draft this paper. X.L., X.Y., J.W., and V.A.L. provided critical technical advice and comments for the experimental studies and manuscript revision. A.Y.L.L. and J.S. provided advise and contributed to the experimental design in collecting cough signals.

## Supporting information

Supporting Information

Supporting Information

Supporting Information

Supplemental Movie 1

Supplemental Movie 2

Supplemental Movie 3

Supplemental Movie 4

## Data Availability

The data that support the findings of this study are available from the corresponding author upon reasonable request.
