# Supplementary material for: Wide‐Bandwidth Nanocomposite‐Sensor Integrated Smart Mask for Tracking Multiphase Respiratory Activities
Source: Adv Sci (Weinh). 2022 Aug 23;9(31):2203565. doi: 10.1002/advs.202203565 (PMC9631096; doi:10.1002/advs.202203565)
Supplement: Supplementary file 1 — Supporting Information [file ADVS-9-2203565-s005.pdf]

ADVANCED SCIENCE
Open Access

Supporting Information

for *Adv. Sci.*, DOI 10.1002/advs.202203565

Wide-Bandwidth Nanocomposite-Sensor Integrated Smart Mask for Tracking Multiphase Respiratory Activities

Jiao Suo, Yifan Liu, Cong Wu, Meng Chen, Qingyun Huang, Yiming Liu, Kuanming Yao, Yangbin Chen, Qiqi Pan, Xiaoyu Chang, Alice Yeuk Lan Leung, Ho-yin Chan, Guanglie Zhang, Zhengbao Yang, Walid Daoud, Xinyue Li, Vellaisamy A. L. Roy, Jiangang Shen, Xinge Yu*, Jianping Wang* and Wen Jung Li**

Supporting Information

A Wide-bandwidth Nanocomposite-Sensor Integrated Smart Mask for Tracking Multiphase Respiratory Activities

Jiao SUO[1, #], Yifan LIU[1,#], Cong WU[1,#], Meng CHEN[1], Qingyun HUANG[1], Yiming LIU[2], Kuanming YAO[2], Yangbin CHEN[3], Qiqi PAN[1], Xiaoyu CHANG[1], Alice Yeuk Lan LEUNG[4], Ho-yin CHAN[1,], Guanglie ZHANG[1], Zhengbao YANG[1], Walid DAOUD[1], Xinyue LI[5], Roy VELLAISAMY[6], Jiangang SHEN[4], XingeYU[2,*], Jianping WANG[3,*] and Wen Jung LI[1,*]*

[1]Dept. of Mechanical Engineering, City Univ. of Hong Kong, Hong Kong, China
[2]Dept. of Biomedical Engineering, City Univ. of Hong Kong, Hong Kong, China
[3]Dept. of Computer Science, City Univ. of Hong Kong, Hong Kong, China
[4]School of Chinese Medicine, The Univ. of Hong Kong, Hong Kong, China
[5]School of Data Science, City Univ. of Hong Kong, Hong Kong, China
[6]James Watt School of Engineering, University of Glasgow, Scotland, UK

#: Equal contribution authors
**: Co-contact authors*

Supporting information includes:

Supplementary Text S1. Theoretical analysis of the effects of different factors on the sensor's sensitivity.

Supplementary Text S2. Theoretical analysis on resonant frequency.

Figure S1. Response of the sensor with 2 wt% and 3wt% CNT to different pressures under different frequencies.

Figure S2. Simulated relationship of sponge porosity and relative resistance change.

Figure S3. Setup of the sponge-based sensor detecting vibrations of different frequencies.

Figure S4. The full width at half maximum (FWHM) of the sensor detects high-frequency vibration signals.

Figure S5. Response of the device to an audio sound of about 58 dB (200 Hz).

Figure S6. Response of the device to an audio sound of 800 Hz.

Figure S7. Comparison of respiratory signals recorded by the 'smart mask' using the oscilloscope and wireless device.

Figure S8. Statistic of gender and age information on the human subjects.

Figure S9. Contact angle (θ) measurement between water and the sponge-based sensor before/after Parylene coated.

Figure S10. Waveforms and spectrograms of different words/phrases recorded by the 'smart mask'.

Figure S11. Resonant characteristic of the CNT/PDMS sponge sensor.

Figure S12. Indentation modulus (E_{IT}) of the CNT/PDMS nanocomposite with different CNT content.

Figure S13. Results of testing the CNT/PDMS sponge structure sensor with a deformable face mask.

Figure S14. Measurement circuit with the freestanding sponge-based sensor using MAX4466 board.

Table S1. Summary of pressure sensitivity characteristics of the CNT/PDMS sponge-based sensor with different parameters.

Table S2. Basic information on the 31 human subjects.

Table S3. The 53 features extracted from each segmented respiratory signal for recognition with SVM.

Table S4 Recognition results of five different CNN models.

Movie S1. Real-time response of the developed smart mask to human respiratory activities (.MP4).

Movie S2. Respiratory sounds of breathing, coughing, and speaking ('robot') (.MP4).

Movie S3. Different words detected by the smart mask (.MP4).

Movie S4. Real-time response of the sensor integrated with a deformable face mask to human respiratory activities (.MP4).

Code S1. SVM (.txt)

Code S2. CNN (.txt)

S1. Theoretical analysis of the effects of different factors on the sensor's sensitivity

The effects of CNT concentration on the sensor's electrical properties and sensitivity are discussed below. The percolation model is commonly used to describe the electrical behavior of composites consisting of conductive filler and polymer (e.g., CNT/PDMS composite). The conductivity of the polymer composite followed a power-law based on percolation theory, as shown in Equation S1:[1]

$$\sigma_{conductivity} = \sigma_0(\phi - \phi_c)^t \qquad (S1)$$

where $\sigma_{conductivity}$ is the conductivity of the composite; σ_0 is a preexponential factor that depends on the conductivity of the fillers, their contact resistance, and the network topology; t is the critical percolation conductivity exponent; ϕ is the filler concentration while ϕ_c is the percolation threshold where a certain filler concentration results in a signification increase in conductance. The conductivity is the reciprocal of resistivity (Equation S2), and the resistivity ($\rho_{resistivity}$) can be expressed by the resistance (R), cross-sectional area (A), and length (L), as shown in Equation S3.

$$\sigma = \frac{1}{\rho_{resistivity}} \qquad (S2)$$

where

$$\rho_{resistivity} = \frac{R * A}{l} \qquad (S3)$$

Combine Equation S1~S3, the conductivity of the polymer composite can be described as Equation S4:

$$\frac{A * R}{l} = \sigma_0(\phi - \phi_c)^t \qquad (S4)$$

For the CNT/PDMS nanocomposite synthesized in this work, the conductivity increases (resistance decreases) significantly when CNT concentration is 3 wt% (Figure 3b) which is considered to be the percolation threshold. The critical exponent t can be estimated by substituting the resistance and concentration value of the nanocomposite with CNT concentration of 4 wt% and 5 wt% into Equation S4, and t is got to be 0.89. The critical exponent depends on the dimensionality of the composite when the material is percolated by a single type of filler. The prediction critical exponent theoretically lies in 1.6~2, while the values obtain experimentally range from 0.7 to 3.1 for CNT-filled composites.[2-7] The value obtained in this experiment (0.89) is a reasonable range. In addition, research has been that the sensitivity would be higher when the conductive filler is at the percolation threshold,[8] which should be 3 wt% in this work.

On the other hand, a model based on the separation change considering various factors (e.g., Young's modulus of the materials, filler particle diameter, potential barrier height, etc) is developed to predict the piezoresistance of the conductor filled polymer.[9] This model assumes the filler is spherical, of the same size, and arranged in a cubic lattice. According to this model, the relative change in resistance of the conductive polymer composite when applied stress can be predicted as the following Equation S5:

$$\frac{|\Delta R|}{R_0} = |\frac{R}{R_0} - 1| = |\left(1 - \frac{\sigma_{stress}}{E}\right) \exp\left\{-\gamma D\left[\left(\frac{\pi}{6}\right)^{\frac{1}{3}} \theta^{-\frac{1}{3}} - 1\right]\frac{\sigma_{stress}}{E}\right\} - 1| \qquad (S5)$$

$$\gamma = \frac{4\pi}{h}\sqrt{2m\varphi} \qquad (S6)$$

By analyzing the Equation, the relative resistance is influenced by the applied pressure (σ_{stress}), matrix Young's modulus (*E*), potential barrier height (φ), filler particle diameter (*D*) and the filler fraction (θ), while *h* is Plank's constant and *m* is electron mass. Considering the porous structure of the sensor in this work, the Young's modulus of the sponge can be estimated by the Equation S7:[10]

$$\frac{E}{E_s} = B\left(\frac{\rho}{\rho_s}\right)^2 \qquad (S7)$$

where E_s is compressive Young's modulus of composite solid (i.e., zero porosity), B is a constant, ρ is the density of sponge and ρ_s is the density of the composite solid. Assume constant volume of the sponge, the density ρ of a sponge with porosity *P* is defined as:[11]

$$\rho = (1 - P)\rho_s \qquad (S8)$$

Thus, the Young's modulus of the structure can be obtained by:

$$E = BE_s(1 - P)^2 \qquad (S9)$$

Therefore, the relationship of the sponge porosity (*P*) and the absolute relative resistance ($|\Delta R/R_0|$) can be investigated, as shown in Figure S2. The plots were obtained with MATLAB and taking the parameter *h* as 4.14E-15 *eV.s*, *m* as 9.1E-28 *g*, φ as 0.5 *eV*,[12] *D* as 10 μm and θ as 0.03. The results show that $|\Delta R/R_0|$ increases with the increasing of the porosity, which proves that the porous structure is helpful for the sensor' sensitivity. Figure S2(a) shows that when the applied stress and porosity are constant, lower Young's modulus results in higher resistance change. Figure S2(b) shows that when the Young's modulus and porosity are constant, higher applied stress induces higher resistance change. Under given conditions, there exists an optimal porosity where $|\Delta R/R_0|$ reaches the maximum, while this porosity varied with the different parameters such as applied stress and Young's modulus. Commercial cube sugars were used as the templates to make the sponge structure in this work, so the porosity is not controlled. However, the

theoretical analysis results can definitely be beneficial for future improve work.

S2. Theoretical analysis on resonant frequency

An object vibrates with largest amplitude under an applied force at the resonant frequency that is close to its natural frequency. For a simple supported solid rectangular plate (rigid body), its natural frequency can be analyzed using Equation (S10):[13]

$$\omega_{mn} = \sqrt{\frac{D_{rigidity}}{\rho t}}\left[\left(\frac{m\pi}{a}\right)^2 + \left(\frac{n\pi}{b}\right)^2\right] \tag{S10}$$

where (m, n) are the mode shape identifier which refer to the number of humps (antinodes) in the plate; ρ is the density; t, a, and b are the thickness, length, and width of the plate; D is flexural rigidity that can be calculated using Equation (S11):

$$D = \frac{Et^3}{12(1-\nu^2)} \tag{S11}$$

where E is the Young's modulus, and ν is the Poisons ratio. Equations (S8) and (S9) can be substituted into the above equation for analyzing the sponge structure developed in this work. Taking (m, n) as (1, 1), which is the simplest model, the natural frequency can be expressed as Equation (S12):

$$\omega = \sqrt{\frac{BE_s t^2(1-P)}{12(1-\nu^2)\rho_s}}\left[\left(\frac{\pi}{a}\right)^2 + \left(\frac{\pi}{b}\right)^2\right] \tag{S12}$$

The equation shows that the natural frequency of the sponge-based sensing structure decreases with decreased plate thickness (with length and width fixed) and increased porosity. A prior work on the vibration analysis of 3D graphene foam circular plate presents the similar conclusion.[14] In addition to the plate model, the equation for calculating the natural frequency for a membrane is similar:[15]

$$\omega = \frac{\alpha}{r^2}\sqrt{\frac{Et^2}{12(1-\nu^2)\rho}} \tag{S13}$$

where α is a constant that is similar to the mode shape identifier, and r is the radius of the circular membrane.

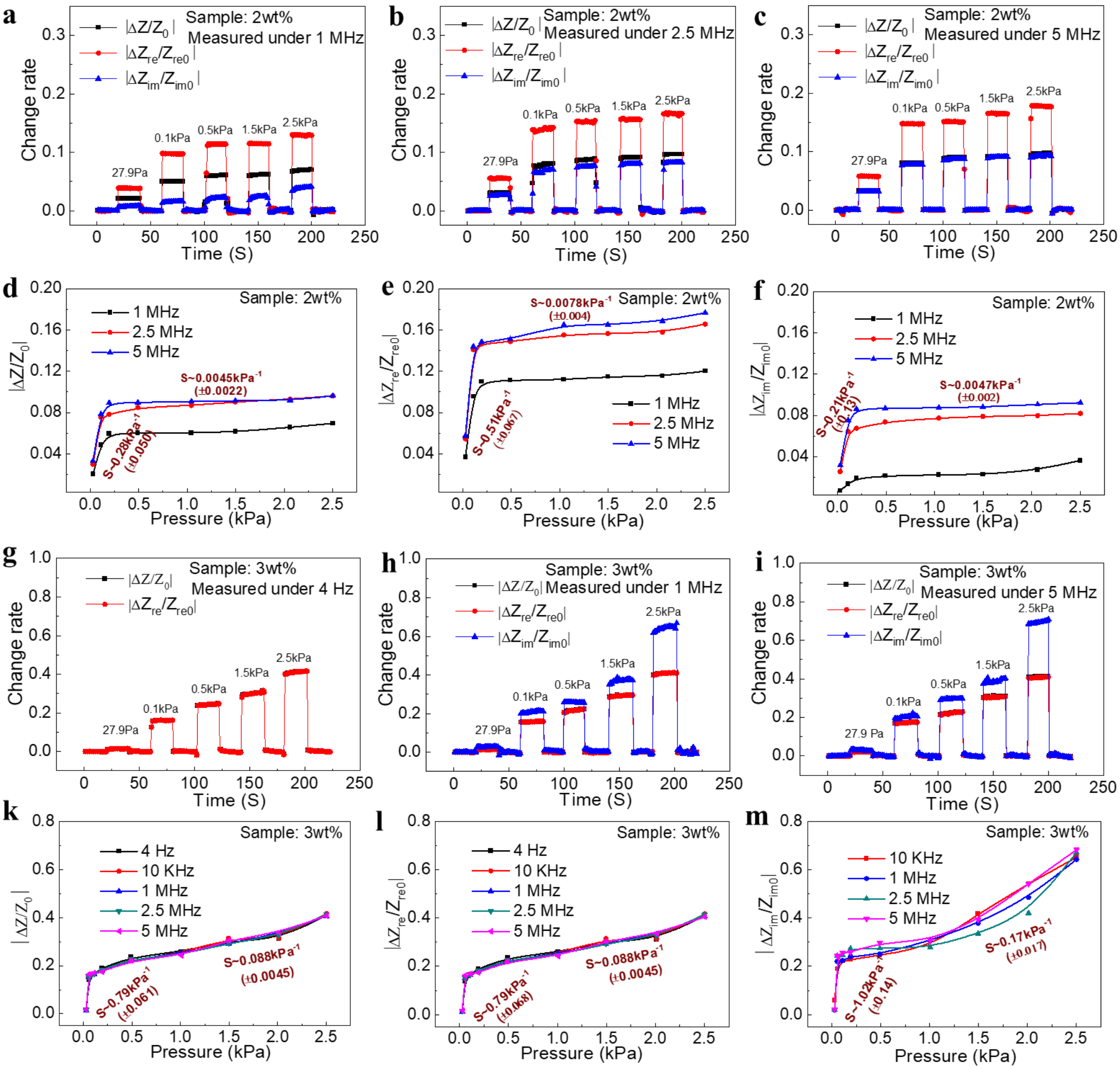

Figure S1. Impedance response of the sensor with 2 wt% and 3 wt% CNT to different pressures under different input electrical current frequencies. (a-c) Change rates and (d-f) the corresponding sensitivities of the sensor with 2 wt% CNT. of Z, Z_{re}, and Z_{im} (Z_0 means at 0 pressure）. (g-i) Change rates and (k-m) the corresponding sensitivities of the sensor with 3 wt% of Z, Z_{re}, and Z_{im}.

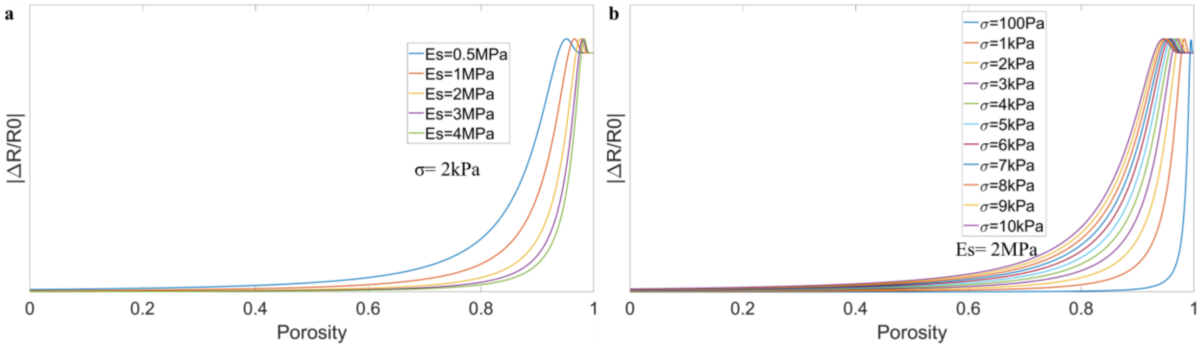

Figure S2. Simulated relationship of sponge porosity and relative resistance change. a) σ_{stress}=2kPa and Es varies from 0.5MPa to 4MPa. b) Es=2MPa and σ varies from 100Pa to 10kPa.

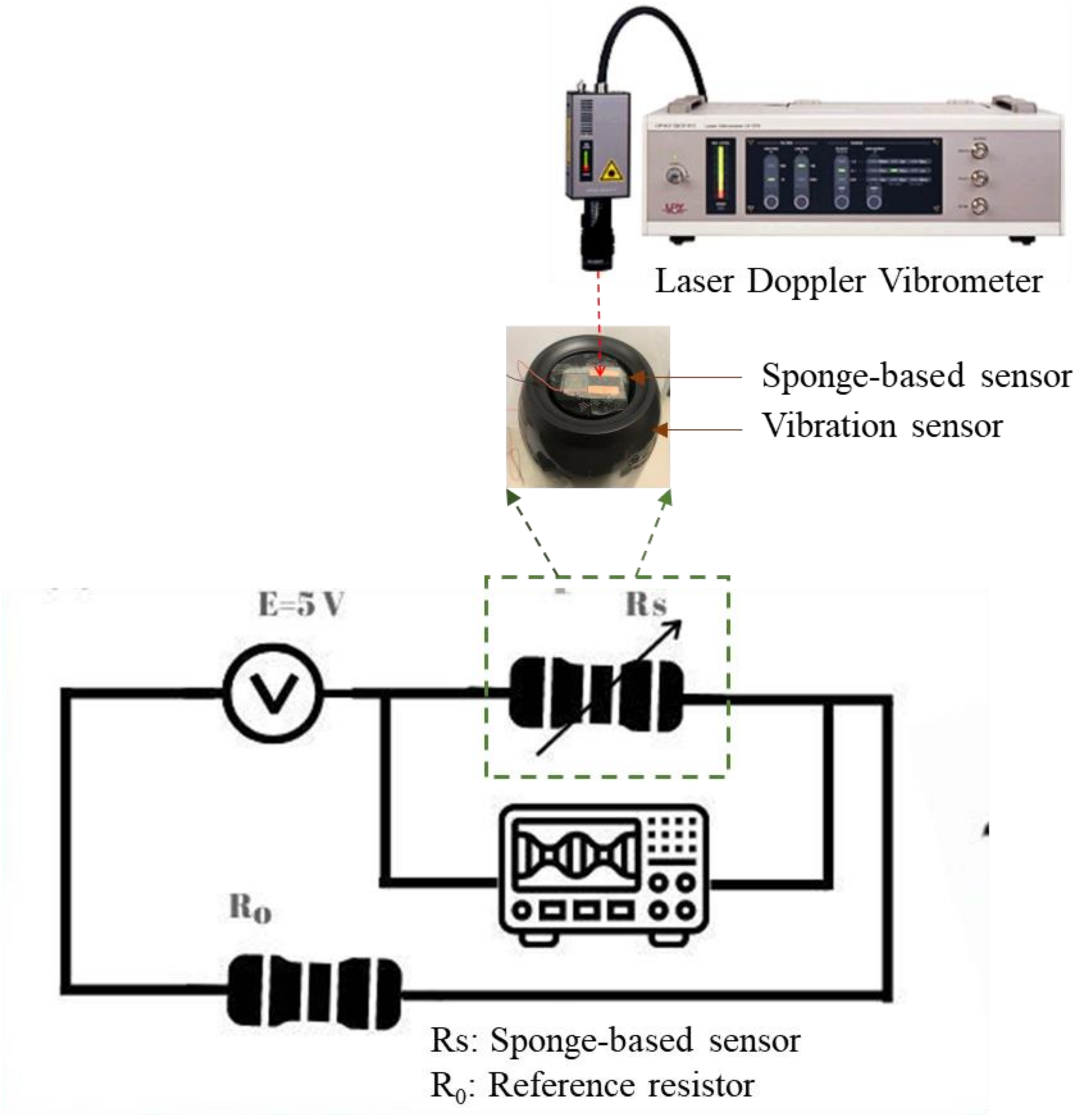

Figure S3. Setup of the sponge-based sensor detecting vibrations of different frequencies.

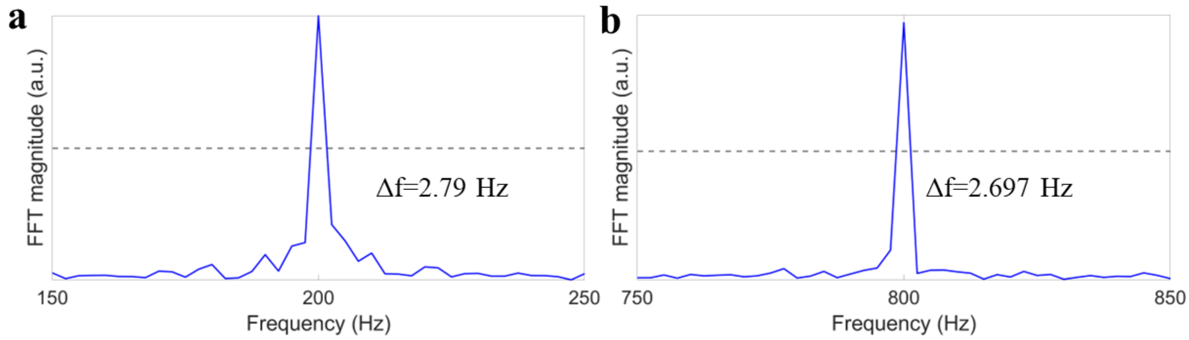

Figure S4. The full width at half maximum (FWHM) of the sensor detecting high frequency vibration signals of (a) 200 and (b) 800Hz.

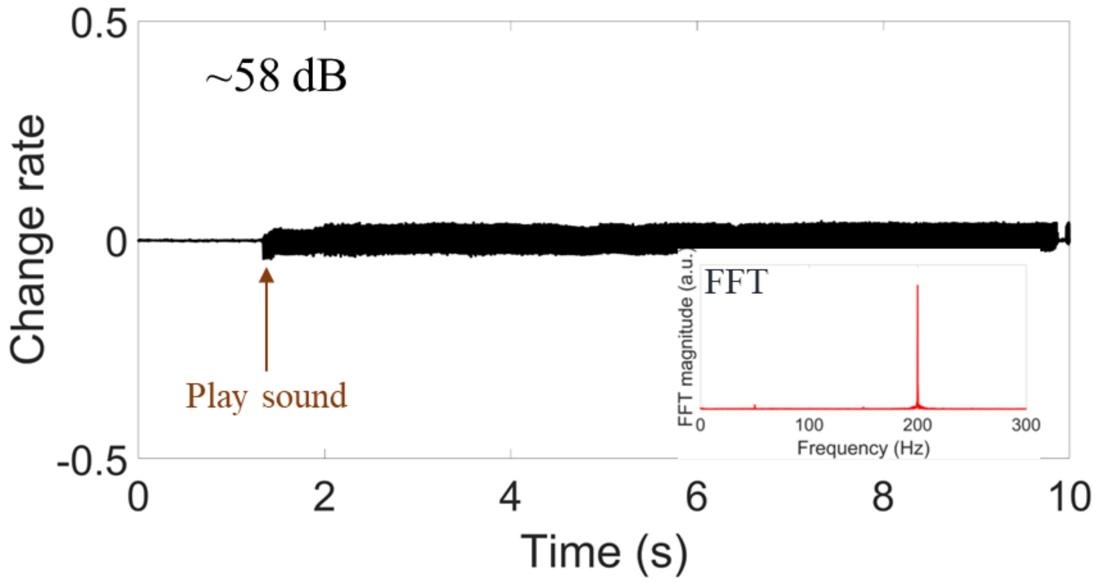

Figure S5. Response of the device to an audio sound of about 58 dB (200 Hz).

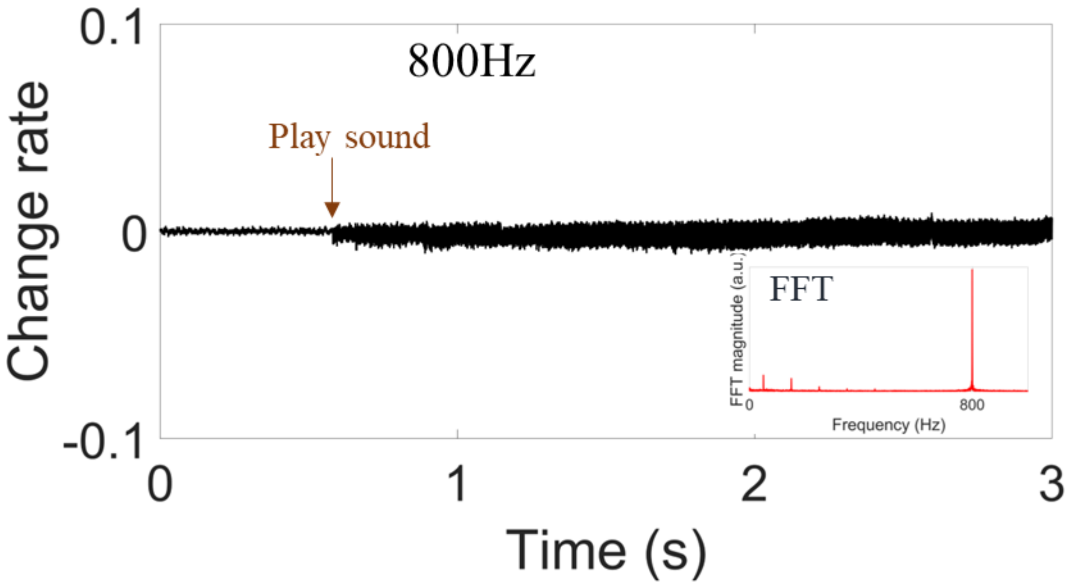

Figure S6. Response of the device to an 800 Hz sound.

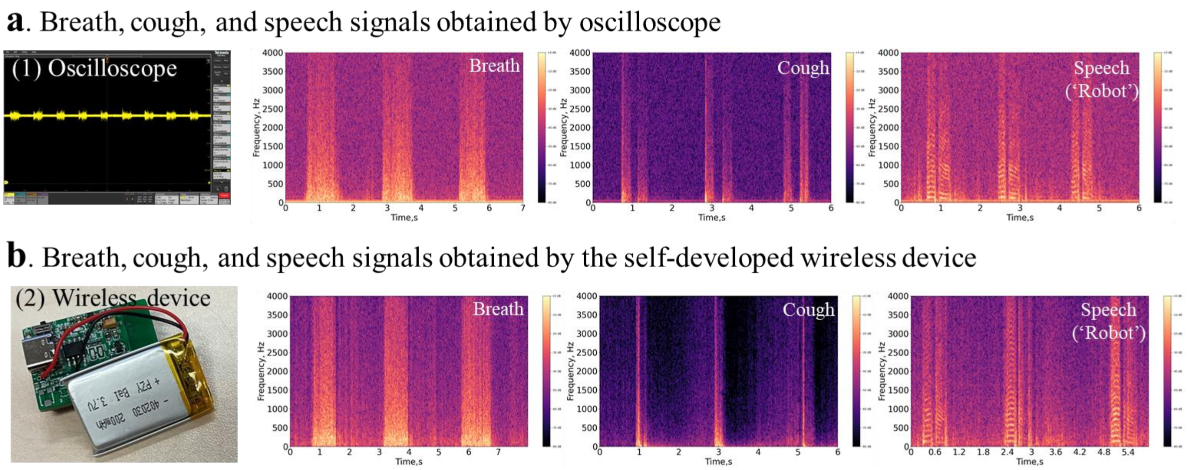

Figure S7. Comparison of respiratory signals recorded by the 'smart mask' using the oscilloscope and wireless device. a) Results from the oscilloscope. b) Results from the self-developed wireless device.

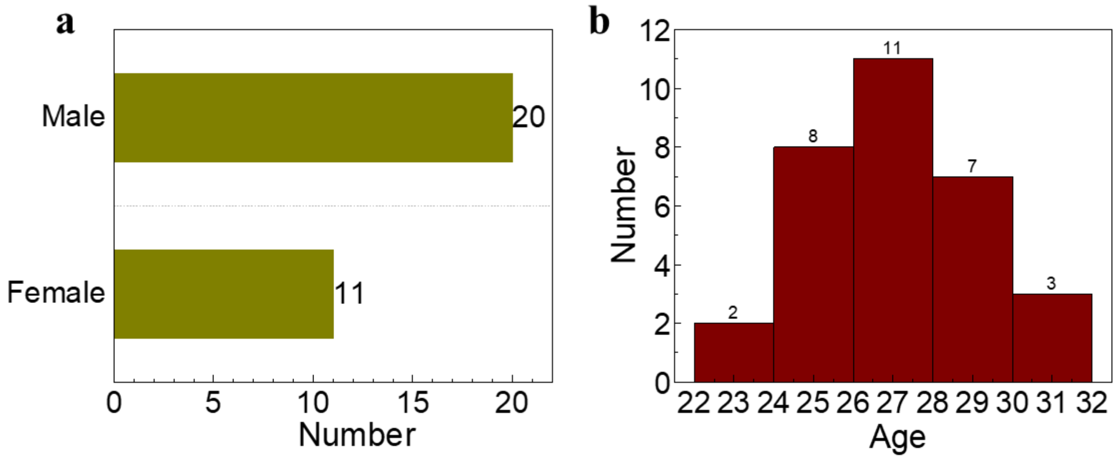

Figure S8. (a) Gender and (b) age information on the thirty-one tested human subjects.

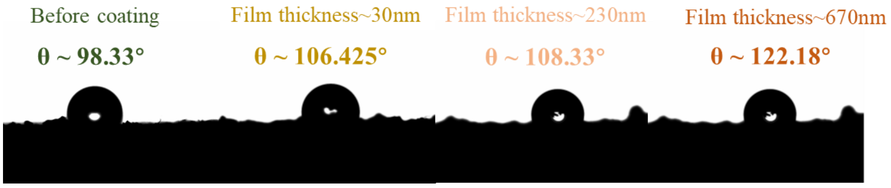

Figure S9. Contact angle (θ) measurement (liquid/volume: water/~5 μL) between water and the sponge-based sensor before/after Parylene coating.

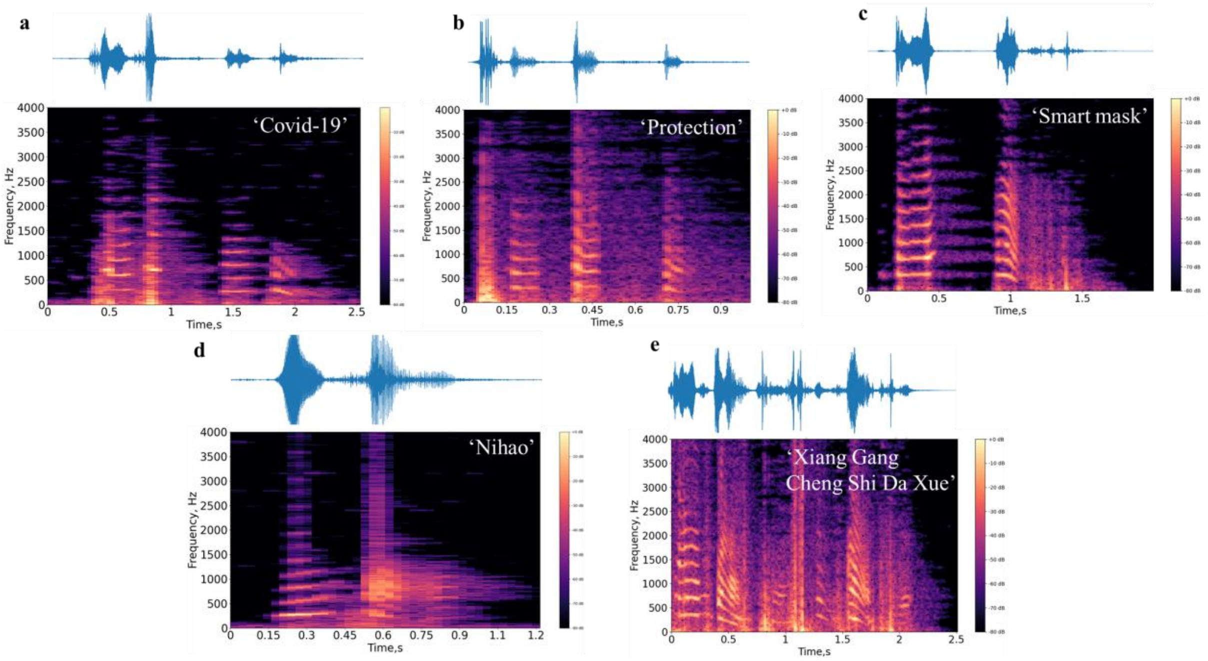

Figure S10. Waveforms and spectrograms of the different words/phrases a) 'COVID-19). B) 'Protection'. c) 'Smart mask'. d) Chinese 'Nihao' e) Chinese 'Xiang Gang Cheng Shi Da Xue' detected by the 'smart mask'

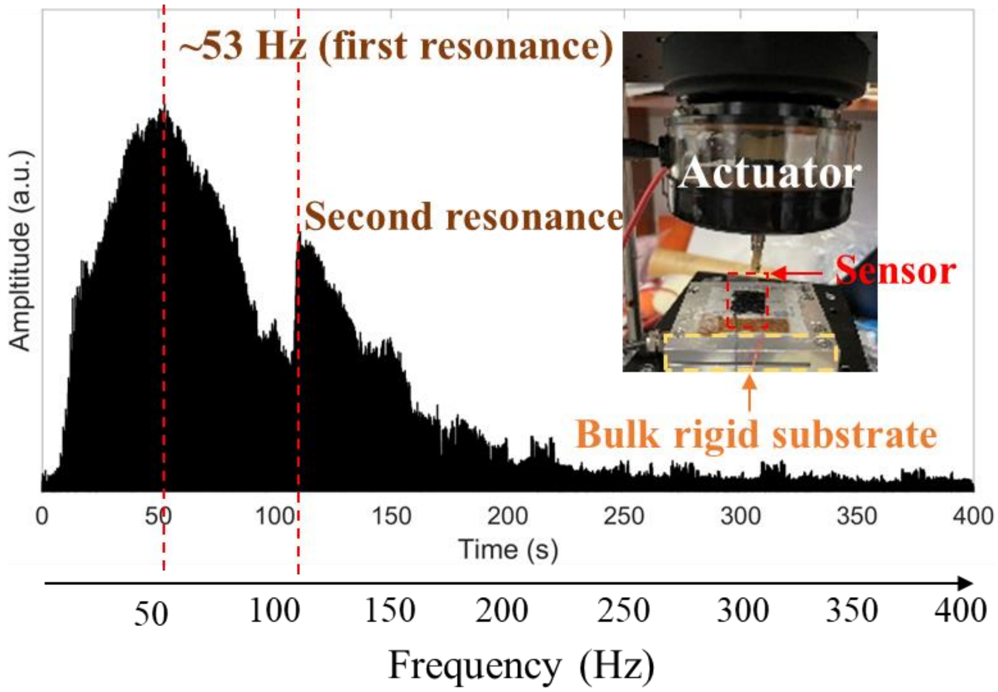

Figure S11. Resonant characteristic of the CNT/PDMS sponge sensor (input frequency increases by 1 Hz per second from 1 Hz to 400 Hz; a.u.: arbitrary units.) The test sample contain 3 wt% CNT, and has a size of 19.6 x 18.4 x 0.4 mm.

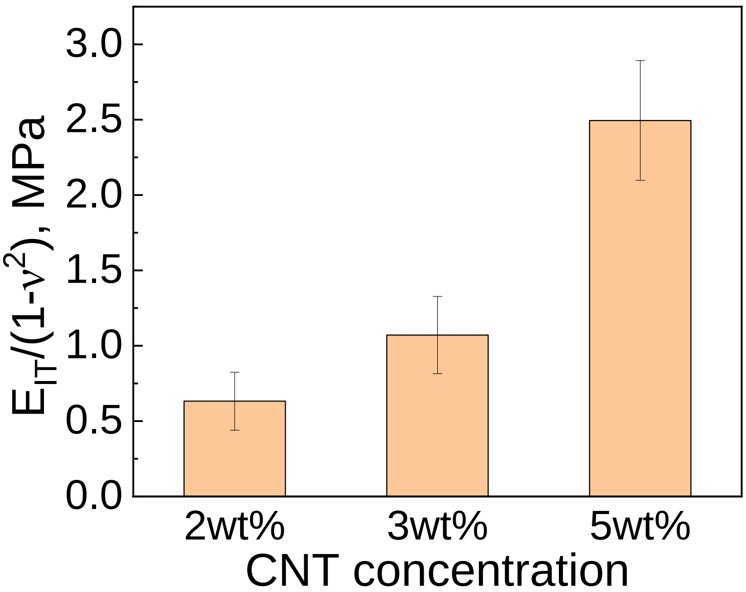

Figure S12. Indentation modulus (E_{IT}, obtained with microhardness test) of the CNT/PDMS nanocomposite with different CNT content.

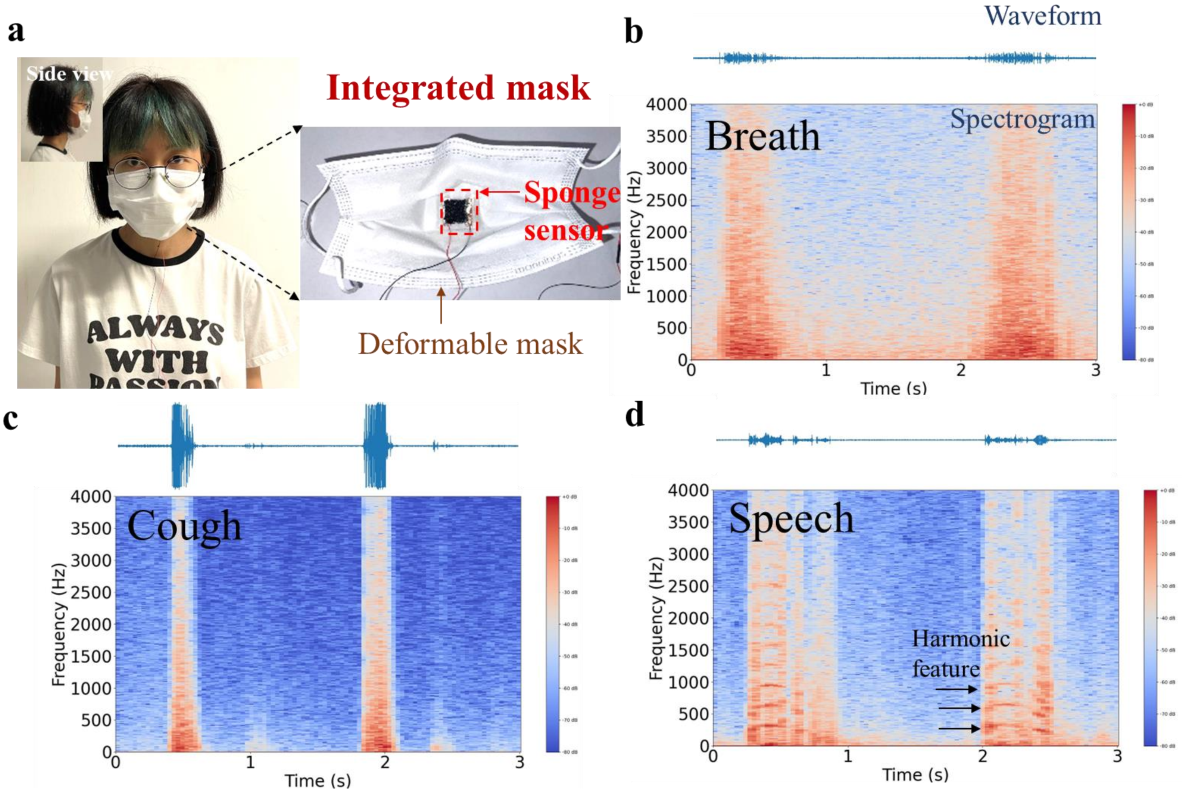

Figure S13. Results of testing the CNT/PDMS sponge structure sensor with a deformable face mask made of polypropylene spunbond non-woven fabric. (a) Picture for the integrated masks and wearing. (b, c, d) Waveforms and spectrograms of the respiratory sounds of breathing, coughing, and speaking.

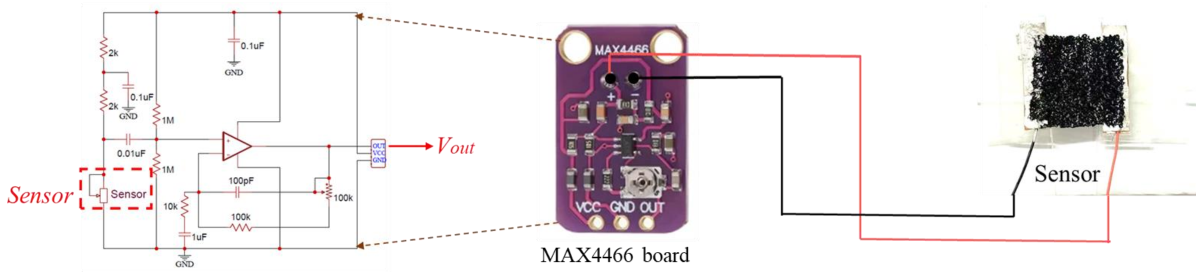

Figure S14. Measurement circuit with the freestanding sponge-based sensor using MAX4466 board.

Table S1. Pressure sensitivity characteristics of total impedance (Z) and its real (Z_{re}) and imaginary (Z_{im}) components for a CNT/PDMS sponge with an approximate thickness of 400 µm and CNT concentrations of 2 wt% and 3 wt%.

Measurement Frequency	Segmented pressure sensitivity of Z, Z_{re}, and Z_{im} (obtained through linear fitting)					
	27.9 Pa–0.2 kPa			0.2–2.5 kPa		
	Z	Z_{re}	Z_{im}	Z	Z_{re}	Z_{im}
2wt% CNT/PDMS sponge-based sensor						
1MHz	0.23	0.43	0.07	0.0042	0.004	0.0063
2.5MHz	0.29	0.55	0.25	0.0069	0.0074	0.0053
5MHz	0.33	0.54	0.32	0.0025	0.012	0.0025
3wt% CNT/PDMS sponge-based sensor						
4 Hz	0.89	0.9	--	0.08	0.08	--
10 kHz	0.78	0.78	0.83	0.09	0.09	0.19
1 MHz	0.78	0.78	1	0.09	0.09	0.17
2.5 MHz	0.8	0.8	1.16	0.09	0.09	0.15
5 MHz	0.72	0.71	1.08	0.09	0.09	0.18

Table S2. Basic information on the 31 human subjects*.

No.	Gender	Age
1	Male	25
2	Female	27
3	Male	24
4	Male	26
5	Female	31
6	Male	24
7	Male	24
8	Female	26
9	Female	24
10	Female	29
11	Male	27
12	Female	27
13	Male	27
14	Male	29
15	Male	24
16	Male	22
17	Male	30
18	Male	26
19	Male	22
20	Female	26
21	Male	29
22	Male	28
23	Male	25
24	Female	27
25	Female	30
26	Male	28
27	Female	27
28	Male	28
29	Female	26
30	Male	28
31	Male	24

* The native languages of the human subjects are Mandarin, Cantonese, Korean, or English.

Table S3. The 53 features extracted from each segmented respiratory signal for recognition with SVM.

53 features	
**Feature**	**Number**
Signal length	1
Mean fundamental frequency	1
Harmonic-noise-ratio (HNR)	1
FFT Max Amplitude	1
Frequency of FFT Max Amplitude	1
Kernel Distribution Estimation (KDE) coefficient	1
Power spectral density (PSD) variance	1
Average spectrum energy	1
Spectrum energy variance	1
Sub-band Spectrum energy (0~250Hz, 250~500Hz, 500~1k Hz, 1k~2k Hz and 2k~4k)	5
Mel Frequency Cepstral Coefficients (MFCCs)	39

Table S4. Recognition results of five different CNN models.

CNN Model name	Model Parameter number	Recall	Precision	F1 score	Accuracy
**AlexNet**	60.9 million	0.9388	0.9380	0.9377	0.9387
**ResNet-18**	33.3 million	0.9399	0.9428	0.9409	0.9416
**VGG-16**	138.4 million	0.9588	0.9531	0.9529	0.9535
**SqueezeNet**	1.2 million	0.9361	0.9365	0.9363	0.9367
**MobileNet-v2**	3.4 million	0.9454	0.9463	0.9458	0.9466

Movie S1. Real-time response of the developed smart mask to human respiratory activities.

Movie S2. Respiratory sounds of breathing, coughing, and speaking (‘robot’).

Movie S3. Different words detected by the smart mask.

Movie S4. Real-time response of the sensor integrated with a flexible face mask to human respiratory activities.

Code S1. SVM

Code S2. CNN

References

[1] D. Stauffer, A. Aharony, *Introduction to percolation theory*, Taylor & Francis, **2018**.

[2] S. M. Yuen, C. C. M. Ma, H. H. Wu, H. C. Kuan, W. J. Chen, S. H. Liao, C. W. Hsu, H. L. Wu, *J. Appl. Polym. Sci.* **2007**, *103* (2), 1272.

[3] O. Regev, P. N. ElKati, J. Loos, C. E. Koning, *Adv. Mater.* **2004**, *16* (3), 248.

[4] C. Martin, J. Sandler, M. Shaffer, M.-K. Schwarz, W. Bauhofer, K. Schulte, A. Windle, *Composites science and technology* **2004**, *64* (15), 2309.

[5] J. Sandler, J. Kirk, I. Kinloch, M. Shaffer, A. Windle, *Polymer* **2003**, *44* (19), 5893.

[6] Z. Ounaies, C. Park, K. Wise, E. Siochi, J. Harrison, *Composites science and technology* **2003**, *63* (11), 1637.

[7] G. Hu, C. Zhao, S. Zhang, M. Yang, Z. Wang, *Polymer* **2006**, *47* (1), 480.

[8] N. Hu, Y. Karube, C. Yan, Z. Masuda, H. Fukunaga, *Acta Mater.* **2008**, *56* (13), 2929.

[9] X. W. Zhang, Y. Pan, Q. Zheng, X. S. Yi, *J. Polym. Sci., Part B: Polym. Phys.* **2000**, *38* (21), 2739.

[10] M. Avalle, G. Belingardi, R. Montanini, *Int. J. Impact Eng.* **2001**, *25* (5), 455.

[11] S. Wang, X. Peng, L. Zhong, J. Tan, S. Jing, X. Cao, W. Chen, C. Liu, R. Sun, *Journal of Materials Chemistry A* **2015**, *3* (16), 8772.

[12] T. G. Kim, U. J. Kim, S. Y. Lee, Y. H. Lee, Y. S. Yu, S. W. Hwang, S. Kim, *IEEE Trans. Electron Devices* **2014**, *61* (6), 2203.

[13] G. N. Guguloth, B. N. Singh, V. Ranjan, *Vibroengineering Procedia* **2019**, *29*, 270.

[14] Y. Q. Wang, M. W. Teng, *Aerospace Science and Technology* **2019**, *95*, 105440.

[15] A. Lima-Rodriguez, J. Garcia-Manrique, W. Dong, A. Gonzalez-Herrera, *Membranes* **2022**, *12* (3), 288.
